# Cisplatin-mediated activation of NF-κB promotes lung cancer stem cell formation via DNA repair pathways

**DOI:** 10.1186/s12967-025-07282-9

**Published:** 2025-11-21

**Authors:** Lingyu Zhang, Qiumei Li, Chunjiang Liu, ShiZhong Wu, Guibin Weng, Ling Wang, Mingshui Chen, Wansong Lin

**Affiliations:** 1 Laboratory of Immuno-Oncology, Clinical Oncology School of Fujian Medical University, Fujian Cancer Hospital, Fuzhou, 350014 China; 2 Fujian Key Laboratory of Translational Cancer Medicine, Fuzhou, 350014 China; 3 College of Chemistry, Fuzhou University, Fuzhou, 350108 China; 4 Department of Radiology, Clinical Oncology School of Fujian Medical University, Fujian Cancer Hospital, Fuzhou, Fujian Province 350014 China; 5 Department of Thoracic Oncology Surgery, Clinical Oncology School of Fujian Medical University, Fujian Cancer Hospital, Fuzhou, 350014 China; 6 The School of Basic Medical Sciences, Fujian Medical University, Fuzhou, 350122 China

**Keywords:** DNA repair, Lung cancer stem cells, DNA-PKcs, NF-κB, Cisplatin resistance

## Abstract

**Background:**

Cisplatin (DDP) an effective DNA-damaging agent, is fundamental in treating non-small cell lung cancer (NSCLC). Resistance to DDP remains a significant challenge in the treatment of NSCLC. This study aimed to elucidate the mechanisms underlying DDP resistance, with a focus on the role of DNA repair pathways and cancer stem cells (CSCs) in NSCLC.

**Method:**

We analyzed p-DNA-PKcs expression in 60 lung cancer tissues (30 DDP-resistant and 30 DDP-sensitive tissues). Using in vitro and in vivo models, such as patient-derived organoids (PDOs) and cell line-derived xenografts, we explored the interplay between DNA repair mechanisms, CSC formation, and NF-κB activation in DDP-resistant NSCLC. The therapeutic potential of targeting DNA-PKcs was also explored using the DNA-PKcs inhibitor NU7441.

**Result:**

Our findings revealed that p-DNA-PKcs is frequently upregulated in DDP-resistant tissues and cell lines and predicts poor prognosis. Activation of the non-homologous end joining (NHEJ) DNA repair pathway by DDP facilitated the stemness of NSCLC. Mechanistically, NF-κB activation was sustained through p300-mediated acetylation of p65 in response to DNA damage, contributing to resistance against DDP. Furthermore, the combination of NU7441 with DDP significantly enhanced the anti-tumor effects in NSCLC models.

**Conclusion:**

This study revealed that NSCLC cells acquire stemness traits through NF-κB activation, with p-DNA-PKcs-induced phosphorylation of p65 being a prerequisite for p65 acetylation and sustained NF-κB activation in drug-resistant cells. Targeting DNA-PKcs represents a novel and effective treatment strategy to overcome DDP resistance in NSCLC.

**Supplementary Information:**

The online version contains supplementary material available at 10.1186/s12967-025-07282-9.

## Background

Lung cancer is a major contributor to cancer-related deaths globally, with NSCLC constituting the majority of cases [1, 2]. Platinum-based chemotherapy, particularly DDP, is the cornerstone of NSCLC treatment. However, DDP resistance significantly compromises its therapeutic efficacy, leading to poor outcomes [3–5]. DDP induces cytotoxicity by causing DNA damage, mainly through intra-strand cross-links that activate DNA repair mechanisms [6]. The robust DNA repair mechanisms in tumor cells, such as non-homologous end joining (NHEJ) and homologous recombination (HR), play a key role in DDP resistance [7, 8]. Unraveling the mechanisms underlying acquired resistance is crucial to preventing lung cancer progression and treatment failure.

Recent studies have shown that DNA repair pathways contribute to DDP resistance and regulate CSCs, a tumor cell subpopulation with self-renewal and differentiation abilities [9]. A growing body of evidence shows that changes in the expression pattern of DNA repair genes can affect the self-renewal ability, tumor-forming potential, and invasiveness of cancer cells, which are key features of CSCs. CSCs, capable of regrowth after treatment, are crucial in developing resistance to chemotherapy, targeted therapy, and immunotherapy [10, 11]. Several subpopulations of CSCs have been identified in NSCLC, including ALDH1A1, CD133, Nanog, and KLF4 [12–14]. Anti-CSC therapies targeting surface markers and pathways across various cancer types have been explored in animal models and clinical trials [15, 16]. However, their efficacy remains limited due to insufficient understanding of the mechanisms underlying DDP-induced enrichment of CSCs.

The NF-κB signaling pathway, alongside DNA repair mechanisms, plays a major role in regulating cancer stemness and chemoresistance [17]. NF-κB is a crucial transcription factor that enhances cell survival, proliferation, and apoptosis resistance after DNA damage [18]. Recent studies have shown that NF-κB activation can enhance the stemness of cancer cells, thereby contributing to treatment resistance and tumor recurrence [19]. The exact mechanisms by which NF-κB engages with DNA repair pathways to enhance cancer stemness in DDP-resistant NSCLC remain unclear.

This study investigated the role of DNA repair pathways and focused on the NHEJ pathway in DDP resistance and CSC formation in NSCLC. Furthermore, we explored the involvement of the NF-κB signaling pathway in this process and unraveled how DNA damage-induced NF-κB activation promotes cancer stemness and chemoresistance. Our study offers novel insights into the molecular mechanisms of DDP resistance and proposes potential therapeutic strategies to overcome DDP resistance in NSCLC.

## Method

### Human tissue samples

Lung cancer tissue samples were obtained from patients at the Department of Thoracic Surgery, Fujian Provincial Tumor Hospital. Demographic and clinical details, including tissue sources for organoid establishment, are summarized in Supplementary Table 1. Patients with NSCLC were diagnosed via imaging and pathological assessment, with tumors staged based on the TNM system. The study protocol was approved by the Ethics Committee of Fujian Provincial Tumor Hospital (Approval Number K2023-118-01), and written informed consent was obtained.

### Establishment and culture of lung cancer organoids

The establishment of lung cancer organoids (LCOs) was conducted strictly following the methods delineated by Kim et al. [20]. Successfully established LCOs were quantified and embedded in 30 µL of Matrigel (356231, Corning) at a concentration of 10,000 crypts/mL. These organoids were then cultured utilizing the LCO kit (K2318-LA, BioGenous).

### Cell lines, compounds

Human non-small cell lung cancer (NSCLC) A549 cells and their DDP-resistant derivatives (designated as A549/DR) were obtained from the Shanghai Cell Bank and authenticated via short tandem repeat (STR) analysis. Cells were cultured at 37 °C in a 5% CO2 incubator using DMEM/F-12 medium supplemented with 10% FBS, 100 units/mL penicillin, and 100 µg/mL streptomycin. NU7441 was purchased from Selleck (TX, USA), and DDP was purchased from MCE.

### ATP assay

The viability of LCOs was measured using the CellTiter-Glo 3D Cell Viability Assay (Vazyme) following the manufacturer’s instructions. The reagent was mixed with organoid culture medium in a 1:1 ratio and luminescence was measured using a multi-plate reader.

### Western blotting

Cells or LCOs were collected and lysed on ice for 20 min using NP-40 lysis buffer supplemented with 1× PMSF, phosphatase, and protease inhibitors. After centrifugation, protein concentrations were measured using the BCA method. Lysate samples (30 µg/lane) were subjected to SDS-PAGE and then transferred to PVDF membranes. Thereafter, 5% dry milk in TBST was used to block the membranes. Next, the membranes were incubated with primary antibodies at 4 °C overnight. After washing, HRP-conjugated secondary antibodies were applied to the bound antibodies and detected using ECL (P0018S, Beyotime, Jiangsu, China) in a BIO-RAD chemiluminescence imaging system (USA).

### Immunoprecipitation

Initially, 10–30 µL of a 50% protein A agarose bead suspension was mixed with 200 µL of the cell lysate (1 mg/mL) and incubated at 4 °C for 0.5-1 h. Subsequently, the lysate was incubated overnight at 4 °C with 5 µg of anti-p65 antibody or normal rabbit IgG antibody as a control. After four washes with 500 µL of the cell lysis buffer, the pellets were resuspended in 20 µL of 3× SDS sample buffer, vortexed, centrifuged, and denatured. The prepared samples were subjected to Western blotting as previously described.

### Antibodies

Supplementary Table 2 provides details on all antibodies utilized in this study.

### Whole-exome sequencing (WES)

DNA from LCOs and their parent tumors was extracted and sequenced using WES at Novogene (Beijing) with Illumina PE150. The data were aligned to the reference genome (GRCh37/hg19/GRCh38) using BWA, and BAM files were generated with Samblaster. Somatic variants were identified using MuTect, and functional predictions were made using ANNOVAR. CNAs were detected by comparing organoids with tumor tissues using Control-FREEC. Mutational signatures were analyzed using the BSgenome package in R.

### RNA-seq

Total RNA was isolated from A549, A549/DR, and A549/DR + NU7441(1µM) cell lines using TRIzol reagent (Invitrogen). Sequencing libraries were prepared by Shanghai Majorbio Bio-pharm Technology and subsequently sequenced on an Illumina HiSeq X Ten platform (150 bp paired-end). After quality control, clean reads were aligned to the reference genome, and gene expression levels were measured as fragments per kilobase of transcript per million mapped reads (FPKM). Differential expression analysis was conducted using edgeR with stringent thresholds (|log2FC| ≥ 1 and FDR-adjusted p-value ≤ 0.05). Functional enrichment of differentially expressed genes (DEGs) was analyzed through KEGG pathway analysis using the clusterProfiler R package.

### LCO and cell viral transduction

Recombinant lentiviral and control vectors were produced by GenePharma (Shanghai, China). For organoid transduction, LCOs were broken down into single cells, exposed to the viral solution for 4 h, then mixed with Matrigel, and cultured for 3 to 7 days with 2 µg/mL puromycin. Cells in 6-well plates were grown to 50% confluence, transduced with virus for 48 to 96 h, and treated with 2 µg/mL puromycin. Stable cell lines expressing the target genes were cultured for further experiments. The target sequences of shRNA are listed in Supplementary Table 3.

### Immunofluorescence

The cells, LCOs, and tissues were fixed, ruptured, and sequentially blocked after treatment. The samples were incubated overnight at 4 °C with primary antibodies against P65, p-P65, and γ-H2AX. After being washed with PBS, the samples were reacted with Alexa Fluor 482 /628/ conjugated secondary antibody (Thermo Fisher Scientific), followed by nuclear-staining with DAPI (1:1000, Beyotime). The fluorescent images were captured using a confocal Microscope.

### Spheroid colony formation

A549 and A549/DR cells were plated at 2000 cells per well in a mixed matrix gel in 24-well plates. Cells were plated at 100 cells per well in a 96-well plate without matrix gel to determine spheroid formation after 2 h of treatment with DDP. After 7 days of incubation in DMEM/F-12 medium, the spheres in individual wells were quantified to measure the self-renewal capacity of cells. Images were taken, and the number of spheres was counted.

### RT-qPCR assay

Using RT-qPCR with specific primers (Supplementary Table 4) and the SYBR Green PCR Master Mix (Life Technologies), the mRNA transcript levels of genes were measured across different cell groups and compared to the control gene GAPDH. TRIzol reagent (Invitrogen, USA) was used to extract RNA, which was then reverse transcribed into cDNA using the PrimeScript RT Reagent Kit (TaKaRa #RR047A). The 2-ΔΔCt method was employed for data analysis.

### HR/NHEJ assay

pDR-GFP, EJ5-GFP, and pCBASceI plasmids were purchased from Addgene (Seoul, South Korea). In NSCLC cells and LCOs containing the pDR-GFP plasmid, temporary expression of the I-SceI enzyme induces a DSB in one of the two mutant GFP genes, SceGFP and iGFP (Figure S2A). Repairing DSBs via HR between mutant GFP genes can restore GFP function, allowing the expression of GFP protein. The EJ5-GFP plasmid contained a GFP-coding sequence interrupted by the puromycin resistance gene (puro), controlled by a distinct promoter. NHEJ repair of I-SceI-induced DSBs reconnected the promoter with the GFP-coding region, thereby restoring the GFP gene (Figure S2B). By quantifying GFP-positive cells, we assessed the efficiency of DSB repair via both HR and NHEJ pathways. NSCLC cells and LCOs were co-transfected with pDR-GFP and EJ5-GFP and then infected with pCBASceI after two days. GFP expression was measured using ImageJ software from NIH, Bethesda, MD, USA.

### Tumorigenicity study

Animal experiments adhered to the guidelines for the care and use of human specimens and animals. The animal experiments were approved by the Institutional Review Board of Fujian Medical University (Approval Number IACUC FJMU 2024 − 0177). Male athymic nude mice were obtained from Fuzhou Nordens Biotechnology Co., Ltd., and A549 cells (300 × 10^6) or A549/DR cells (2 × 10^6) were injected subcutaneously into their right flanks. In the A549 model, once tumor size reached approximately 100 mm³, mice were divided into two groups (*n* = 5 per group) for treatment with either vehicle (CTL, PBS) or DDP (4 mg/kg, i.p.) every two days for 17 days. In the A549/DR model, mice with a tumor size of 100 mm³ were allocated into four groups (*n* = 5 per group) and received a vehicle (CTL, PBS), DDP (4 mg/kg, i.p., alternate days), NU7441 (10 mg/kg, i.p., alternate days), or the combination of DDP and NU7441. Tumor volumes were measured using the formula [(width)² × (height)]/2. Tumor size and mouse weight were recorded every other day. At the end of the study, the mice were euthanized, and their tumors were dissected for Western blotting to assess the expression levels of p-P65, CD133, SOX9, KLF4, Nanog, and ALDH1A1. The TUNEL assay was used to analyze cell apoptosis in tumor tissues.

### In vitro DNA-PKcs kinase assay

The DNA-PKcs kinase activity was measured using an in vitro reconstitution system as previously described [21] with some modifications. Briefly, recombinant wild-type (WT) or mutant (S536A) p65 protein (1 µg, purified from E. coli) was incubated with immunoprecipitated DNA-PKcs in kinase buffer (125 mM Tris–HCl, pH 7.9, 125 mM MgCl2, 5 mM DTT, and 50% glycerol) containing 0.16 μm [γ-³²P]ATP (PerkinElmer), 20 nM KU70/80, and 100 ng sheared salmon sperm DNA (as DNA-PK activator). Reactions (20 µL final volume) were conducted at 30 °C for 30 min and terminated by adding 5× SDS loading buffer.

### Plasmids and transient transfection

All recombinant plasmids, including the wild-type Flag-tagged p65 and its mutant p65-S536A (TCC→GCC), were synthesized by GenePharma (Shanghai, China). For transient transfection, A549/DR cells were seeded in 150 mm culture dishes and transfected with 30 µg of the target plasmid using Lipo3.0 transfection reagent (Hanheng Biotechnology) following the manufacturer’s instructions. After 48 h of transfection, the cells were harvested for subsequent analyses. Successful transfection was confirmed by Western blotting of Flag-tagged protein.

### Immunohistochemistry (IHC) staining and quantification

Formalin-fixed, paraffin-embedded (FFPE) tumor tissue sections, with a thickness of 4 μm, underwent a process of deparaffinization and rehydration, followed by antigen retrieval utilizing a citrate buffer at pH 6.0. Endogenous peroxidase activity was inhibited using a 3% hydrogen peroxide solution. Subsequently, the sections were incubated overnight at 4℃ with primary antibodies targeting phosphorylated DNA-PKcs, KU80, and KU70, at dilutions detailed in Supplementary Table 2. Post-washing, the sections were treated with a horseradish peroxidase (HRP)-conjugated secondary antibody. Diaminobenzidine (DAB) served as the chromogen, and hematoxylin was employed for counterstaining.

IHC scoring was conducted by two independent pathologists who were blinded to the patient groups. The evaluation of staining involved an assessment of both the intensity of staining and the percentage of positively stained cells. Staining intensity was graded on a scale from 0 to 2, where 0 indicated negative staining, 1 indicated weak staining, and 2 indicated strong staining. A modified H-score was calculated for each sample using the formula: Modified H-score = [(percentage of weakly stained cells × 1) + (percentage of strongly stained cells × 2)]. This calculation yields a theoretical range from 0 to 200. For graphical representation and statistical analysis, the raw modified H-score values were normalized by dividing by 100, resulting in a score range from 0 to 2. Based on these normalized scores, samples were classified into low expression and high expression groups for subsequent statistical analysis.

### Statistical analysis

Each experiment was conducted three times. Data are presented as mean ± standard deviation (SD). Using GraphPad Prism 8.0, student’s t-test was conducted to assess differences between control and experimental groups. *P* ≥ 0.05 was deemed non-significant (ns) (*P < 0.05, **P < 0.01 and ***P < 0.001).

## Results

### DDP activated DNA repair via the NHEJ pathway in lung cancer cells

**Fig. 1:**
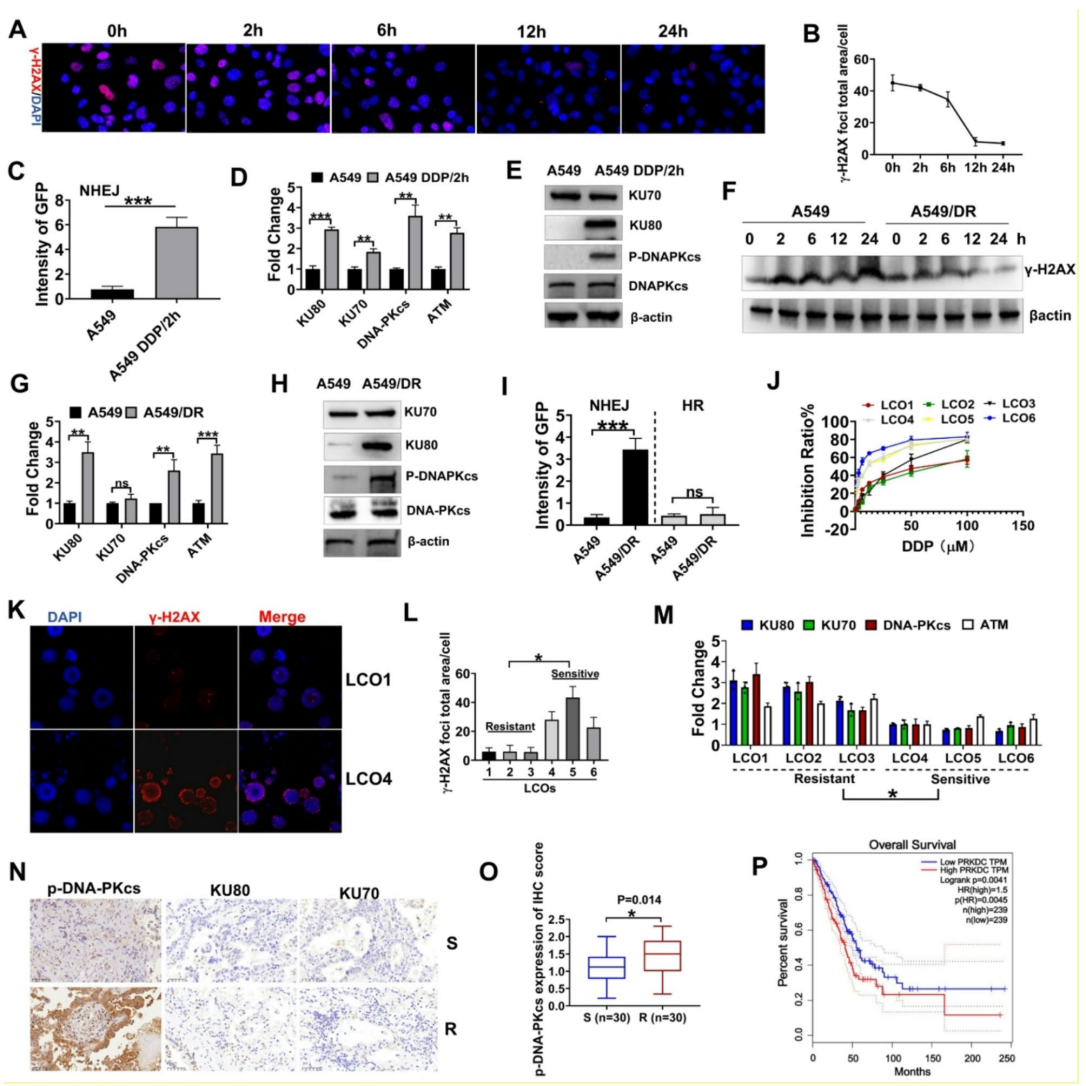
DDP-resistant cells showed enhanced DNA repair via the NHEJ pathway. **A** Immunofluorescence assay of γ-H2AX in A549 cells after 2 h of treatment with DDP and staining at various time points. **B** Quantification of γ-H2AX levels in cells from three independent experiments. **C** GFP intensity of A549 cells after the repair of I-SceI-induced DSBs via NHEJ, measured using EJ5-GFP assays. Cells were exposed to either a vehicle or 4 µM DDP. DDP/2 h refers to A549 cells being stimulated with DDP for 2 h, followed by the removal of the drug. **D** Analysis of HR and NHEJ gene expression in A549 cells using qRT-PCR. **E** Western blotting of KU70, KU80, p-DNA-PKcs, and DNA-PKcs in A549 cells after treatment with vehicle or 4 µM DDP. **F** Western blotting for γ-H2AX expression in A549 and A549/DR cells over time after treatment with DDP. **G** qRT-PCR was employed to measure the expression of NHEJ genes in A549 and A549/DR cells. **H** Western blotting was conducted to measure the expression of the related proteins in A549 and A549/DR cells. **I** The intensity of GFP in A549 and A549/DR cells, after being repaired by HR or NHEJ. **J** Cell proliferation in LCOs after treatment with DDP (72 h) was assessed via ATP assay. **K** High-content imaging of γ-H2AX at 200× magnification. **L** Measurement of γ-H2AX levels in LCOs. **M** qRT-PCR was conducted to measure the mRNA expression levels of KU70, KU80, DNA-PKcs and ATM in LCOs. **N** IHC staining and IHC intensity of KU70, KU80, and p-DNA-PKcs in DDP-sensitive tumors and DDP-resistant tumors (*N* = 30). **O** IHC staining of p-DNA-PKcs. **P** In the Kaplan-Meier curve, the dotted line indicates the 95% CI. HR, hazard ratio; In the NCBI database, DNA-PKcs and PRKDC refer to the same protein. “S” indicates DDP sensitivity, while “R” indicates DDP resistance. *** *P* < 0.001; ** *P* < 0.01; * *P* < 0.05; ns, not significant

To measure DNA damage response in DDP-resistant lung adenocarcinoma, A549 cells were exposed to DDP for 2 h (using the optimized dose determined in Figure S1). This treatment transiently upregulated γ-H2AX, a well-established biomarker of DNA double-strand breaks (DSBs) [22]. The subsequent decrease in γ-H2AX levels after removing DDP demonstrated an efficient DNA repair response in these cells (Fig. 1A-B). Reporter assays (DR-GFP/EJ5-GFP) revealed that a brief DDP treatment (for 2 h) followed by drug withdrawal significantly enhanced NHEJ repair activity in A549 cells (Fig. 1C, Figure S2A-C).

Analysis of mRNA levels revealed significant upregulation of the mRNA levels of core NHEJ components (KU70, KU80, and DNA-PKcs), while key HR factors (BRCA1, RAD51) [23] remained unchanged and PARP1 showed only a slight increase (Fig. 1D, Figure S2D). Western blotting confirmed markedly increased phosphorylation of NHEJ-related proteins (DNA-PKcs and KU80) (Fig. 1E). Protein expression of HR components (BRCA1, RAD51) remained unchanged, with only marginal upregulation of PARP1 (Figure S2E). Concurrent upregulation of the upstream regulator ATM [24] was also observed (Fig. 1D). Collectively, these results suggest that DDP-induced DNA damage is primarily repaired through the NHEJ pathway.

In DDP-resistant A549/DR cells, γ-H2AX levels remained consistently high but decreased after 24 h of treatment with DDP, suggesting increased DNA repair capacity (Fig. 1E-F, Figure S2F). This coincided with a marked upregulation of NHEJ components, notably DNA-PKcs and KU80, alongside an elevation in ATM levels(Figure 1G-H). In contrast, HR-related genes and proteins, such as BRCA1 and RAD51, exhibited no significant change (Figure S2G-H). While there was a slight increase in PARP1 expression, this was considerably less pronounced compared to the upregulation observed in NHEJ components (Figure S2H). NHEJ/HR assay also revealed that the NHEJ activity was abnormally elevated in A549/DR cells (Fig. 1I).

We established six lung cancer patient-derived organoids (PDOs) with varying degrees of sensitivity to DDP to validate these findings in a clinically relevant model. These PDOs closely mirrored the primary tumor characteristics and confirmed the heterogeneous responses to DDP in patients (Figures S3-4, Fig. 1J). For example, patient 4 exhibited significant tumor size reduction on CT scans after treatment with DDP, while patient 1 exhibited tumor progression and resistance, consistent with the results of the PDO sensitivity test (Figure S5).

The organoids were exposed to DDP for 2 h, and γ-H2AX levels in organoids were measured 12 h later. Organoids from DDP-sensitive patients (LCO4-6) showed high γ-H2AX staining, indicating ongoing DNA damage and repair. In contrast, organoids from resistant patients (LCO1-3) exhibited decreased γ-H2AX staining, suggesting more efficient DNA repair (Fig. 1K-L, Figure S6). Additionally, drug-resistant organoids showed significantly higher expression of NHEJ repair-related genes compared to sensitive ones (Fig. 1M). Immunohistochemical (IHC) staining of tumor samples from 60 patients revealed higher levels of phosphorylated DNA-PKcs and KU80 in DDP-resistant tumors compared to DDP-sensitive ones (Fig. 1N-O, Figure S7A). Further analysis revealed that p-DNA-PKcs expression was not associated with patients’ age or sex, but it was positively associated with lymph node metastasis, distal metastasis, and tumor grade (Supplementary Tables 1^1^ and 5). Moreover, elevated levels of DNA-PKcs were associated with poor prognosis among patients with NSCLC (Fig. 1P, Figure S7B), suggesting its potential role in mediating DDP resistance.

### DDP-induced DNA repair promotes CSC formation in NSCLC

Studies have indicated that CSCs possess superior DNA repair capabilities compared to non-stem cells, which contributed to their chemoresistance [25]. Recent studies have also highlighted the role of DNA-PKcs in mediating treatment resistance in CSCs [26]. Based on these findings, we next measured the effect of DDP-induced DNA damage on CSC characteristics, focusing on the timing of DNA repair that could enhance stemness of NSCLC cells.

**Fig. 2:**
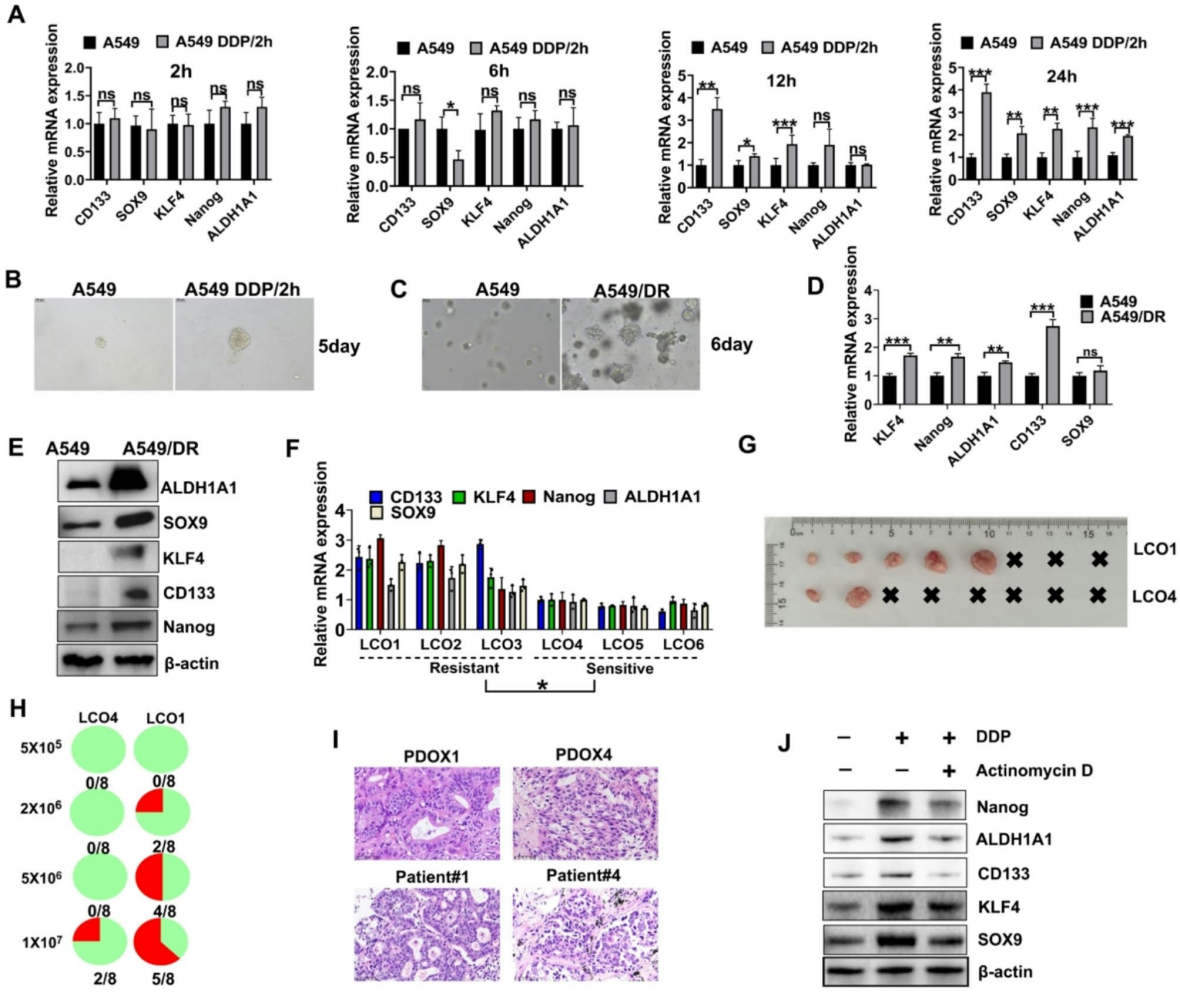
The link between DNA repair and CSC formation. **A** Two hours after drug removal, RT-PCR was used to measure the expression of stemness-related genes in A549 cells at various time points. **B** Formation of tumorspheres from individual tumor cells. DDP/2 h refers to A549 cells being stimulated with DDP for 2 h, followed by the removal of the drug. **C** Tumorsphere formation of A549 and A549/DR. **D** The mRNA expression levels of stemness genes in A549 and A549/DR cells. **E** Western blotting was conducted to assess the expression levels of ALDH1A1, SOX9, KLF4, CD133, and Nanog in A549 and A549/DR cell lines. **F** RT-PCR analysis was conducted to measure the mRNA expression levels of ALDH1A1, SOX9, KLF4, CD133, and Nanog in LCOs. **G** Representative images of tumor formation in vivo for LCO1 and LCO4. **H** The tumorigenic rates of LCO1 and LCO4 in vivo with different cell numbers. **I** H&E staining of PDOX1 and PDOX4, which are PDO-based xenografts derived from LCO1 and LCO4, respectively, as well as their corresponding patient tissues. **J** The expression levels of stemness genes in A549 cells treated with chemotherapy and actinomycin D. A549 cells were exposed to DDP for 2 h, followed by 24 h of treatment with the DNA repair inhibitor actinomycin D. *** *P* < 0.001; ** *P* < 0.01; * *P* < 0.05; ns, not significant

We first measured the expression of stemness-related genes in A549 cells at various time points after treatment with DDP. The results of RT-PCR revealed a significant increase in the expression of stemness genes, including ALDH1A1, SOX9, KLF4, CD133, and Nanog, 24 h after exposure to DDP (Fig. 2A). This change was accompanied by a marked increase in tumor sphere formation, indicating enhanced self-renewal capacity (Fig. 2B). DDP-resistant A549/DR cells generated a higher number of tumor spheres than DDP-sensitive A549 cells, underscoring the significance of DNA repair in CSC enrichment (Fig. 2C).

We measured the mRNA expression of stem cell-related genes in A549/DR cells to assess the association between DNA repair and CSC formation. RT-PCR analysis revealed a notable upregulation in gene expression (Fig. 2D). Western blotting also confirmed these results, showing increased protein levels of stemness markers in A549/DR cells compared to A549 cells (Fig. 2E).

We analyzed LCOs with varying degrees of sensitivity to DDP to validate these results in a more clinically relevant model. Consistent with our cell line data, drug-resistant organoids exhibited significantly higher expression levels of stemness markers compared to DDP-sensitive organoids (Fig. 2F). Moreover, LCO1 cells from a DDP-resistant patient exhibited enhanced potential for tumorigenesis in vivo compared to LCO4 cells derived from a DDP-sensitive patient (Fig. 2G-I).

We treated A549 cells with DDP followed by actinomycin D, an inhibitor of DNA repair to determine whether DNA repair, rather than DNA damage-induced mutations, is responsible for CSC formation. Western blotting demonstrated a notable decrease in the protein levels of stemness markers in cells treated with actinomycin D compared to the controls (Fig. 2J). These findings suggest that DNA repair processes, rather than mutations caused by DNA damage, are critical for the stemness of NSCLC cells after chemotherapy.

#### NF-κB activation via p300-mediated p65 acetylation sustains cancer stemness in NSCLC

**Fig. 3:**
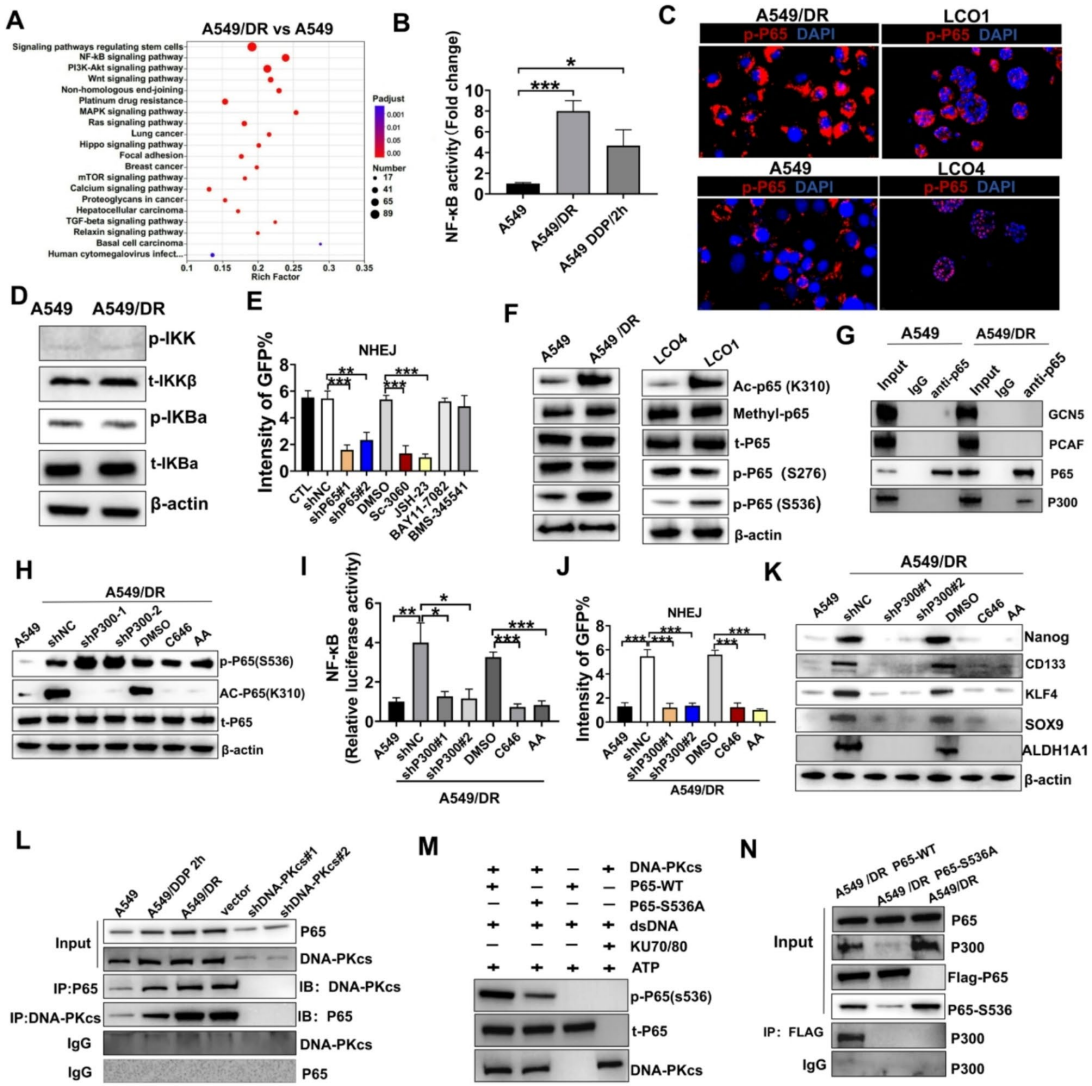
The activation of NF-kB via P300-mediated acetylation of P65 helped sustain the stemness of drug-resistant cells. **A** KEGG pathway analysis identifies highly expressed signaling pathways in A549/DR. The analysis was performed using three A549/DR and A549 samples. **B** Luciferase reporter assays demonstrated NF-kB activity. **C** Representative immunostaining of p-P65 in LCOs and A549 cell lines. **D** Western blotting was conducted to measure the total and phosphorylated levels of IKK and IkBa in A549 and A549/DR. **E** Intensity of GFP in A549/DR Cells Repaired by NHEJ under different conditions. The “CTL” group represents untreated A549/DR cells serving as a control. **F** Western blotting was conducted to assess the expression levels of total p65 and its Lys310 acetylation, methylation, and phosphorylation at Ser536 and Ser276 in specified A549 cells and LCOs. **G** The interactions of p65 with HAT molecules (GCN5, PCAF, and p300) in A549 and A549/DR cells were measured using immunoprecipitation. **H-K** A549/DR cells were transduced either with non-targeting control shRNA (shNC) or with p300-targeting shRNA, or pretreated with p300 inhibitors (C646 or anacardic acid [AA]), using DMSO as the vehicle control. **H** Western blotting was used to measure the phosphorylation of Ser536 and the acetylation of Lys310 on p65. **I** Luciferase reporter assays were conducted to measure NF-κB activity. **J** The intensity of GFP in cells repaired by NHEJ was measured using EJ5-GFP reporter assays. **K** The Nanog, CD133, KLF4, SOX9, and ALDH1A1 were measured by Western blotting. **L** Co-IP analysis of the interaction between P65 and DNA-PKcs in A549, A549/DDP 2 h and A549/DR cell lines under different conditions. **M** In vitro assessment of DNA-PKcs kinase activity was conducted to evaluate its ability to phosphorylate p65 at the S536 residue. Purified DNA-PKcs was incubated with either P65-WT or a P65-S536A in the presence of the KU70/80 heterodimer and dsDNA using ATP as the phosphate donor. The reaction mixtures were subsequently separated via SDS-PAGE and analyzed through immunoblotting. **N** CO-IP experiment to assess the interaction between P300 and p65 in A549/DR cells expressing either wild-type (WT) or the Ser536Ala (S536A) mutant of p65. *** *P* < 0.001; ** *P* < 0.01; * *P* < 0.05; ns, not significant

Although the association between CSCs and chemoresistance has been previously identified, the molecular mechanisms involved in CSC maintenance remain unclear. Previous studies have implicated several signaling pathways, including PI3K/AKT, WNT, NOTCH, and NF-κB, in the regulation of CSC phenotype and chemoresistance [27]. Among them, NF-κB has emerged as a key player in mediating DDP resistance in NSCLC [28]. Our sequencing data also revealed significant enrichment of the NF-κB signaling pathway in A549/DR cells (Fig. 3A).

To investigate the role of p65 in DDP resistance, we analyzed its expression in tissues with varying DDP sensitivity. As a key subunit of NF-κB, p65 regulates NF-κB activity through both canonical and non-canonical pathways [28]. Immunofluorescence assays revealed significantly higher p-P65 expression in DDP-resistant tissues than in DDP-sensitive tissues (Figure S8). Consistently, enhanced transcriptional activity of NF-κB was observed in both A549/DR and A549 cells after 2 h of treatment with DDP, which persisted after drug removal (Fig. 3B). Furthermore, increased nuclear translocation of p65 was detected in A549/DR cells, LCO1s(Fig. 3C). Interestingly, although NF-κB activity was enhanced in A549/DR, the phosphorylation levels of IKK and IκB-key components of the canonical NF-κB pathway remained unchanged compared to A549 cells (Fig. 3D). This finding suggested that NF-κB activation in A549/DR cells may occur through alternative mechanisms. We investigated the role of NF-κB in NHEJ-mediated DNA repair using NF-κB nuclear translocation inhibitors (Sc-3060 and JSH-23) and p65 shRNA. These interventions successfully decreased DNA-PKcs phosphorylation, highlighting the critical role of NF-κB in this repair process (Figure S9 A-B). IKK inhibitors (BAY-117082 and BMS-345541) did not affect NHEJ activity, suggesting that NF-κB activation in DDP-resistant cells circumvents the canonical IKK/IκB pathway (Fig. 3E). To investigate the mechanisms underlying increased NF-κB activity in these cells, we analyzed post-translational modifications of the p65 protein, as previous studies have indicated that its methylation and acetylation facilitate nuclear retention [29].

Western blotting revealed a significant increase in p65 acetylation at K310 in A549/DR cells, with no changes in its methylation (Fig. 3F). We measured p65 phosphorylation since p65 acetylation is contingent upon its phosphorylation [30]. p65 phosphorylation at S536 was significantly enhanced in A549/DR, despite low phosphorylation levels of IKK or IkB, whereas S276 phosphorylation was not upregulated (Fig. 3F). This phenomenon was also verified in organoids (Fig. 3F). This finding suggests that NF-kB activation in DDP-resistant cells, characterized by p65 phosphorylation and acetylation, is maintained through alternative signaling pathways instead of IKK activation.

Immunoprecipitation with a p65 antibody identified p300 as the histone acetyltransferase responsible for p65 acetylation in A549/DR cells, an interaction not observed in A549 cells (Fig. 3G). Silencing p300 with shRNA or pharmacological inhibitors significantly downregulated p65 acetylation without affecting its phosphorylation (Fig. 3H). Furthermore, p300 inhibition suppressed NF-kB activation (Fig. 3I) and NHEJ activation (Fig. 3J). This finding suggests that p300-mediated acetylation of p65 is critical for sustaining NF-κB signaling and DNA repair in DDP-resistant cells. We investigated the effects of p300 inhibition on CSC characteristics and found that p300 downregulation in A549/DR cells significantly decreased the expression of stemness markers (Fig. 3K). Similar effects were observed in organoids, where p300 inhibition in LCOs lowered the expression of stemness-related genes and DNA repair markers (Figure S10A-B).

The upstream kinase responsible for initiating the phosphorylation cascade of p65 at Ser536 remains unknown. Since DNA-PKcs is activated by DDP-induced DNA damage and exhibits structural homology with IKK kinases [31], we hypothesized that DNA-PKcs may function as the initiating kinase in this signaling pathway. We measured the role of DDP-activated DNA-PKcs in mediating p65-S536 phosphorylation independently of IKK to investigate this hypothesis. Co-immunoprecipitation assays demonstrated a direct interaction between DNA-PKcs and p65 in A549/DR cells (Fig. 3L). Additionally, in vitro kinase assays revealed that DNA-PKcs specifically phosphorylated wild-type p65 (p65-WT) but not the S536A mutant (Fig. 3M), thereby confirming that DNA-PKcs directly phosphorylates p65 at Ser536. Combined analysis using the p65-S536A mutant and Co-IP demonstrated that p300-mediated acetylation at K310 strictly relies on phosphorylation at S536 (Fig. 3N).

Together, these findings delineate a complete signaling axis where DDP-induced DNA damage activates DNA-PKcs. DNA-PKcs then phosphorylates p65-S536 to enable p300 recruitment and K310 acetylation. This process not only sustains NF-κB activity but also promotes DNA repair through the NHEJ pathway, thereby promoting CSC formation and chemoresistance in NSCLC.

### P65 phosphorylation induced by p-DNA-PKcs was found to be a prerequisite for its acetylation and NF-κB activation in DDP-resistant cells

**Fig. 4:**
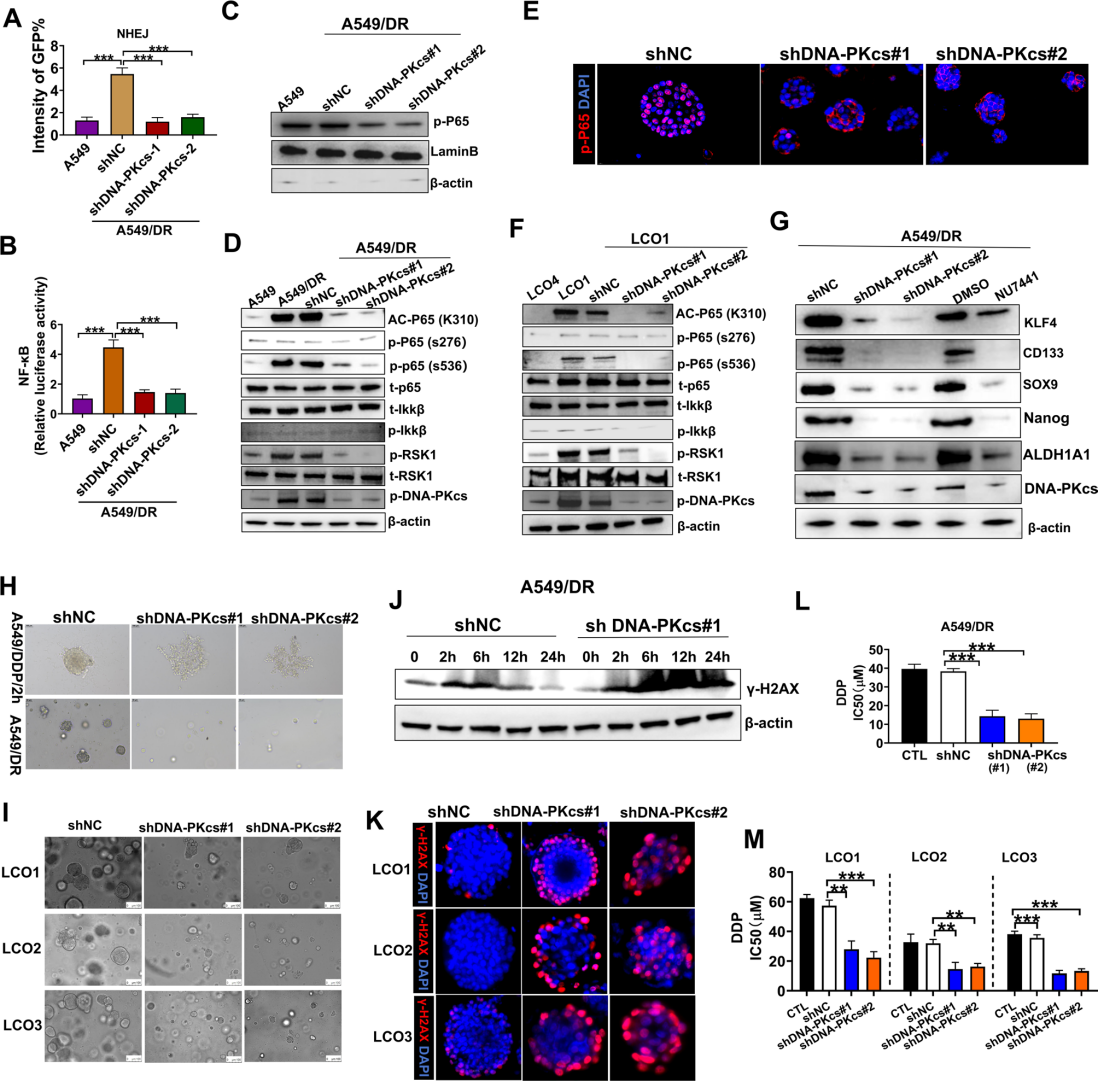
p-DNA-PKcs-induced phosphorylation of P65 was essential for its subsequent acetylation and the maintenance of NF-κB activation in A549/DR cells. **A-F** show A549/DR and LCO1 cells were transduced with either shNC or DNA-PKcs-targeting shRNA. **A** The intensity of GFP in A549/DR cells repaired by NHEJ determined using EJ5-GFP reporter assays. **B** Luciferase reporter assays revealing NF-kB activity. **C** Western blotting of nuclear p-p65 protein in A549/DR cells. **D** Western blotting of A549/DR cells to detect specified proteins. **E** Immunofluorescent images of LCO1 illustrating the translocation of p65 nuclear at 400X magnification. **F** Western blotting of specified proteins in LCO1. **G** Western blotting for specified proteins. A549/DR cells were transduced with shNC or DNA-PKcs shRNA, or pretreated with DNA-PKcs inhibitors (NU7441), considering DMSO as a negative control. **H** Downregulation of DNA-PKcs followed by the assessment of cell spheroid formation. DDP/2 h refers to A549 cells stimulated with DDP for 2 h, followed by drug removal. **I** Representative images of LCOs after DNA-PKcs knockdown. **J** Western blotting was used to detect γ-H2AX levels. **K** Detection of γ-H2AX in LCO1-3 through immunofluorescence. **L-M** IC50 of DDP in A549/DR cells (**L**) and LCOs (**M**) after downregulation of DNA-PKcs. *** *P* < 0.001; ** *P* < 0.01; * *P* < 0.05; ns, not significant

We examined if the upregulation of KU80 or p-DNA-PKcs in the NHEJ pathway activates NF-κB in DDP-resistant NSCLC, aiming to clarify DNA-PKcs’s role in NF-κB signaling and its impact on DDP resistance. In A549/DR cells, the knockdown of DNA-PKcs, but not KU80, notably suppressed NHEJ activity, downregulated the transcriptional activity NF-κB, and reduced the nuclear retention of p65 (Figs. 4A-C and S11). This was accompanied by decreased S536 phosphorylation and K310 acetylation of p65, but S276 phosphorylation remained unchanged (Fig. 4D). The LCO1 model confirmed these findings, showing that DNA-PKcs knockdown decreased the nuclear retention of p65, S536 phosphorylation, and K310 acetylation, but did not affect S276 phosphorylation (Fig. 4E-F).

Building on the results of Chen et al. [32]. regarding the critical role of S536 phosphorylation in the K310 acetylation of p65, we investigated the interactions among IKK, RSK1, and DNA-PKcs in phosphorylating p65. Compared to A549 cells, RSK1 phosphorylation was significantly upregulated in A549/DR cells, which was completely inhibited by DNA-PKcs silencing (Fig. 4D). This finding was consistent with those from organoid models (Fig. 4F), indicating that DNA-PKcs-mediated phosphorylation of p65 is crucial for its acetylation and prolonged NF-κB activation in drug-resistant cells.

Furthermore, the downregulation of DNA-PKcs in A549/DR cells significantly impaired their ability to enrich CSCs, as evidenced by the reduced expression of CSC markers, including KLF4, CD133, SOX9, Nanog, and ALDH1A1 (Fig. 4G), and a significant decrease in the sphere formation ability of A549/DR (Fig. 4H). The down-regulation of DNA-PKcs in A549 cells also resulted in the elimination of their sphere-forming ability following a 2-hour treatment with DDP (Fig. 4H). Moreover, downregulation of DNA-PKcs reduced levels lowered mRNA of CSC markers and inhibited PDO growth (Fig. 4I, S12A-D). These findings suggest that DNA-PKcs plays a pivotal role in regulating CSC enrichment and chemoresistance, likely by modulating NF-κB signaling.

Compared to the control group, A549/DR cells with DNA-PKcs knockdown continued to show strong γ-H2AX staining 12 h after treatment with DDP (Figs. 4J). Moreover, in LCO1-3 cells derived from drug-resistant patients, γ-H2AX was significantly enhanced after DNA-PKcs knockdown (Fig. 4K). This finding indicates that DNA damage repair is impaired due to the absence of DNA-PKcs.

Importantly, the resistance of A549/DR cells to DDP was partly reversed after DNA-PKcs knockdown, evidenced by decreased IC50 values (Fig. 4L). Similarly, the downregulation of DNA-PKcs reinstated the sensitivity of LCO1-3 cells to DDP (Fig. 4M), suggesting that DNA-PKcs acts as a critical regulatory enzyme for DNA repair and chemoresistance in CSCs. These findings also highlight NF-κB activation as a key mediator involved in DNA-PKcs-induced chemoresistance, a core pathway that we propose in the graphical abstract.

#### Pharmacological inhibition of DNA-PKcs enhanced the anti-tumor effects of DDP in vitro

**Fig. 5:**
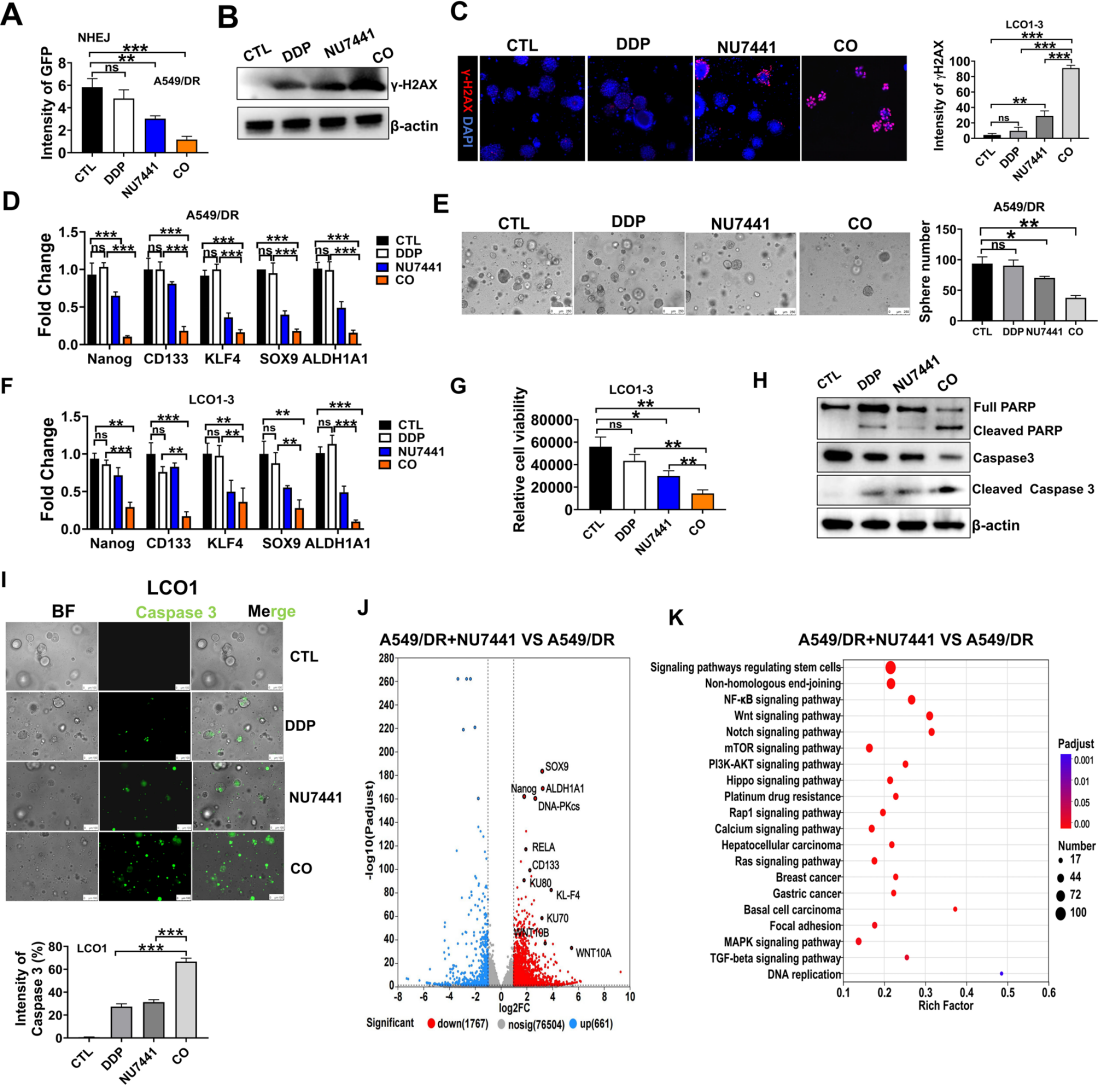
Pharmacological inhibition of DNA-PKcs enhanced the anti-tumor effects of DDP. **A** The percentage of GFP-positive cells repaired via the NHEJ pathways after treatment with vehicle, 4 µM DDP, 1 µM NU7441, alone or combined (CO). **B** γ-H2AX levels in A549/DR cells after treatment with DDP and NU7441, measured by Western blotting. **C** Immunofluorescence analysis showing γ-H2AX levels in LCOs after treatment with 10 µM DDP and 2 µM NU7441 based on intensity data from three experiments. **D** RT-PCR was employed to measure the mRNA levels of ALDH1A1, SOX9, KLF4, CD133, and Nanog in A549/DR cells. Cells were exposed to vehicle, 4 µM DDP, 1 µM NU7441, or their combination. **E** Co-treatment with DDP and NU7441 inhibited spheroid colony formation in A549/DR cells. The right panel presents the quantification of spheroid colony formation. **F-G** LCO1-3 was exposed to a vehicle, 10 µM DDP, 2 µM NU7441, or their combination (CO). **F** RT-PCR was employed to measure mRNA levels in LCO1-3. The cells were treated with vehicle, DDP, NU7441, or their combination. **G** The viability of LCO1-3 was measured using an ATP assay. **H** Western blotting was conducted on A549/DR cells to measure the levels of full PARP, cleaved PARP, cleaved caspase-3, and caspase-3 after exposure to vehicle, 4 µM DDP, 1 µM NU7441, or their combination for 2 days. **I** Immunofluorescence assay indicated that treatment with NU7441 augmented caspase3 expression in LCO1 exposed to DDP. CO indicates the co-treatment with DDP and NU7441. **J** The volcano plot of DEGs between A549/DR and A549/DR + NU7441 (*n* = 3 biologically independent samples per group); Red and blue dots represent downregulated and upregulated genes, respectively (FDR < 0.05; |Log2(fold change)| >1). Representative downregulated genes pivotal to the studied pathways (e.g., NF-κB signaling, stemness, and DNA repair) are explicitly labeled and highlighted with a black border. **K** KEGG enrichment analysis of genes significantly downregulated in A549/DR + NU7441 compared to A549/DR (*n* = 3 biologically independent samples per group). Bubble color: Adjusted P-value; Bubble size: Number of genes in the pathway (Count). *P < 0.05; **P < 0.01; ***P < 0.001; ns, not significant

In our study, we employed NU7441, a highly effective and selective inhibitor of DNA-PKcs [33], to explore the function of DNA-PKcs in DNA damage response and stemness and determine its potential clinical applications. Our findings demonstrated that treatment with 1 µM NU significantly hindered NHEJ (Fig. 5A). Western blotting revealed that NU7441 significantly enhanced DDP-induced DNA damage in A549/DR cells, evidenced by γ-H2AX clearance (Fig. 5B). Furthermore, immunofluorescence analysis indicated that the combination of NU7441 with DDP increased γ-H2AX levels in LCOs (Fig. 5C). Our study indicated that inhibition of DNA-PKcs can effectively block DNA repair pathways. We systematically evaluated the effects of PARP1 inhibition (targeting HR) in combination with NU7441 to determine whether chemoresistance is specifically driven by NHEJ pathway activation or involves compensatory HR mechanisms. Strikingly, in A549/DR cells, the combination of DDP with both NU7441 and PARP1 inhibitor increased γ-H2AX levels comparable to DDP + NU7441 dual therapy alone (Figure S13A-B). Importantly, the triple combination (DDP + NU7441 + PARP1) showed no significant additional benefit over the dual combination (DDP + NU7441) in terms of growth inhibition (Figure S13C). This observation was consistently replicated in LCO models (Figure S13D). Most notably, assessment of stemness markers revealed that inhibition of the HR pathway did not enhance NU7441-mediated suppression of CSC properties (Figure S13E-F), suggesting that DDP resistance primarily relied on NHEJ activation without substantial HR pathway compensation.

Furthermore, we discovered that a low dose of NU7441 significantly reduced CSC enrichment in drug-resistant cancer cells, as shown by decreased expression of key markers of CSCs (ALDH1A1, KLF4, CD133, Nanog, and SOX9) (Fig. 5D) and diminished sphere formation ability (Fig. 5E). Similar effects were observed in LCO models (Fig. 5F-G). These findings indicated that DNA-PKcs specifically affects DNA repair processes in CSCs.

Given that silencing DNA-PKcs enhanced the sensitivity of A549/DR cells to DDP, we explored whether pharmacological inhibition of DNA-PK can modulate drug resistance. Treatment with both DDP and NU7441 increased the levels of cleaved PARP and cleaved Caspase3 in A549/DR cells, suggesting enhanced apoptosis (Fig. 5H). Interestingly, this combination synergistically enhanced Caspase3 expression in LCOs (Fig. 5I, Figure S14A-D).

We performed RNA-seq analysis in A549, A549/DR, and NU7441-treated A549/DR cells to elucidate the transcriptional regulatory mechanism underlying DNA-PKcs inhibition. Strikingly, A549/DR cells exhibited significant upregulation of CSC-associated genes (e.g., ALDH1A1, SOX9, KLF4, CD133, and Nanog), which was reversed upon DNA-PKcs inhibition by NU7441 (Fig. 5J). The volcano plot further highlights the concomitant downregulation of pivotal genes across functionally distinct pathways, including core components of NHEJ such as KU70 and KU80, key NF-κB signaling molecules like RELA, and putative stemness-related transcriptional targets of p65, namely WNT10A and WNT10B. These findings were corroborated by KEGG enrichment analysis, which demonstrated that DNA-PKcs inhibition concurrently suppresses pathways related to NF-κB signaling, DNA damage repair, and cellular stemness (Fig. 5K). Furthermore, bioinformatic analysis using the JASPAR database predicted WNT10A, WNT10B, and other effector genes as direct transcriptional targets of p65 (Supplementary Table 6), thereby establishing a direct mechanistic link between NF-κB activation, dysregulated DNA repair, and the maintenance of the cancer stem cell phenotype.

Altogether, these findings establish DNA-PKcs inhibition as a promising strategy to overcome DDP resistance in NSCLC through selective disruption of NHEJ-mediated DNA repair, abrogation of NF-κB-driven stemness programs, and potentiation of apoptosis without HR pathway compensation.

#### In vivo evidence for targeting DNA-PKcs to overcome DDP resistance in NSCLC

**Fig. 6:**
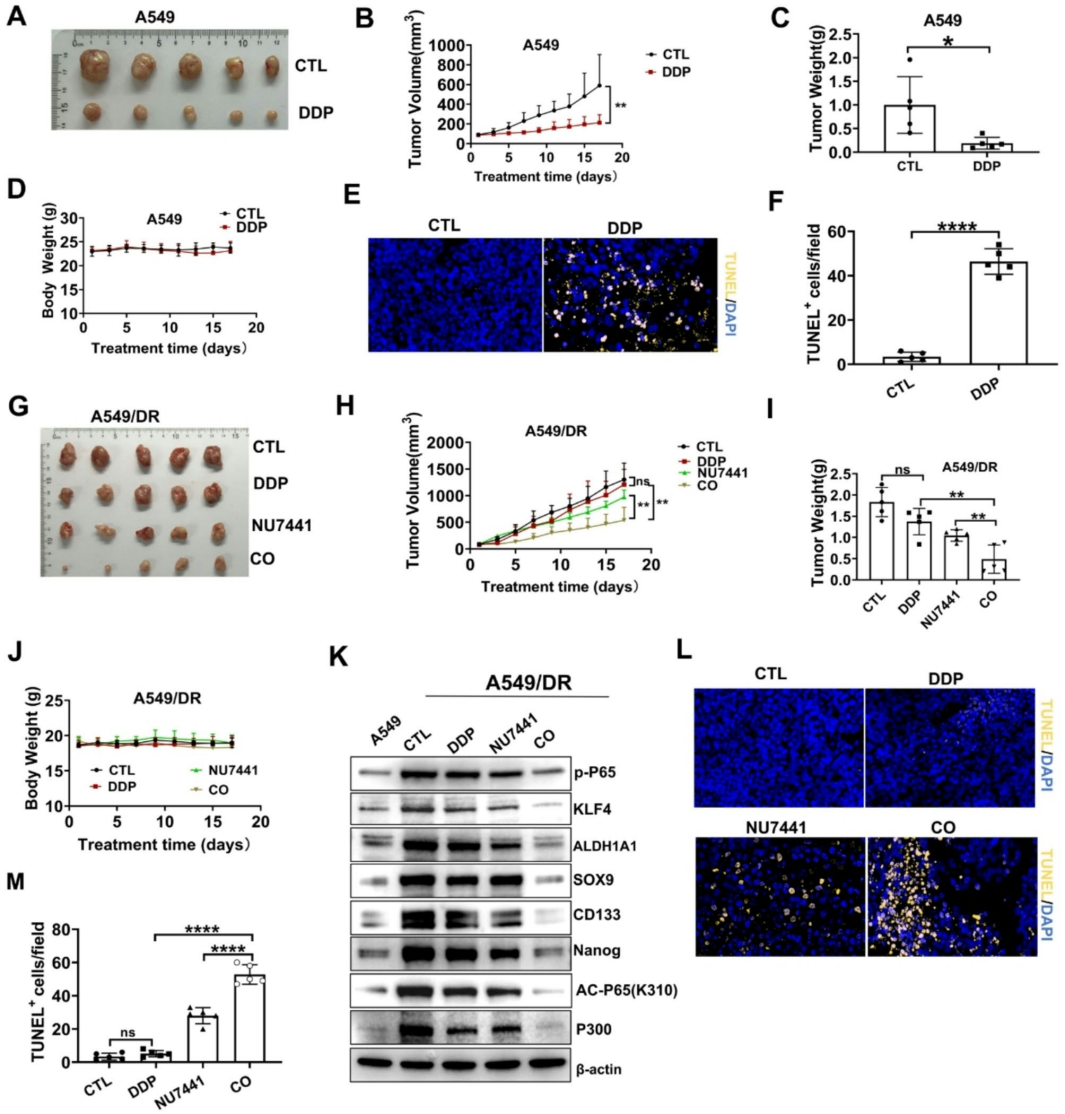
Targeting DNA-PKcs to overcome DDP resistance in NSCLC (in vivo evidence). **A** Image showing xenograft tumors in mice implanted with A549 cells and treated with DDP. **B** The dynamic growth of different groups of xenograft tumors (*n* = 5 per group). **C** Tumor weight was measured in mice with xenografts. **D** Changes in the body weight of mice. **E-F** TUNEL analysis of apoptotic cells in pare xenograft tumors. **E** TUNEL analysis of apoptotic cells in pare xenograft tumors. (Magnification x 200). **F** Quantitative analysis of TUNEL-positive cells per field. **G** An image of all xenograft tumors in mice implanted with A549/DR and treated with the indicated drugs. **H** The dynamic growth of different groups of A549/DR tumors in mice (*n* = 5 per group). **I** Tumor weight was measured in mice bearing A549/DR xenografts. **J** Changes in the body weights of mice. **K** Western blotting was conducted to measure the relative protein levels of p-P65, Nanog, KLF4, CD133, SOX9, P300 and AC-P65(K310) in A549/DR tumors. **L**-**M** TUNEL analysis of apoptotic cells in resistant Xenograft Tumors. **L**TUNEL analysis of apoptotic cells in xenograft tumors (Magnification x 200). **M** Quantitative analysis of TUNEL-positive cells per field. CO indicates the co-treatment with DDP and NU7441. *P < 0.05; **P < 0.01; ns, not significant

Based on in vitro findings, we pharmacologically inhibited DNA-PKcs to enhance the antitumor efficacy of DDP in vivo. We established mouse xenograft models utilizing both A549 and A549/DR cell lines. In the A549 model, treatment with DDP significantly reduced tumor growth (Fig. 6A-C). After 17 days of treatment, body weight remained unchanged (Fig. 6D). The TUNEL assay indicated that DDP effectively induced tumor cell apoptosis (Fig. 6E-F). Conversely, A549/DR tumors, characterized by their resistance to DDP, exhibited a minimal response to DDP monotherapy. However, the combination of NU7441 with DDP offered a marked antitumor response (Fig. 6G-I). Importantly, no significant alterations were observed in body weight 17 days after treatment (Fig. 6J).

In the A549/DR xenograft tumor model, the combination of DDP and NU7441 significantly reduced the levels of p-p65 in tumor tissues, while concurrently suppressing K310-mediated acetylation of p65 and the expression of the histone acetyltransferase P30 (Fig. 6K). Western blotting also demonstrated a significant decrease in the expression of stemness markers ALDH1A1, KLF4, Nanog, SOX9, and CD133 in the solid tumors of mice treated with the combination of NU7441 and DDP (Fig. 6K). This improved tumor response was associated with increased tumor cell apoptosis (Fig. 6L-M). These data demonstrate that targeting DNA-PKcs can overcome DDP resistance triggered by NF-κB activation.

## Discussion

CSCs are a resilient tumor subpopulation with self-renewal and differentiation capabilities, which can promote tumor recurrence [34]. This study revealed that DDP can upregulate the NHEJ pathway-related gene p-DNA-PKcs, enhancing DNA repair and promoting CSC formation in NSCLC. Our findings indicated that NF-κB activation, facilitated by DNA-PKcs-induced phosphorylation and acetylation of p65, is crucial in enhancing cancer stemness and chemoresistance.

Recent findings suggested that increased expression of DNA repair genes in CSCs enhances their resistance to treatment by improving DNA repair mechanisms. In line with these findings, our study demonstrated that treatment with DDP upregulated p-DNA-PKcs, enhancing NHEJ-mediated DNA repair and promoting CSC formation in NSCLC. Using organoids from DDP-sensitive and DDP-resistant patients, along with the A549 cell line, we showed that short exposure to DDP (2 h) significantly enhanced NHEJ capacity. Furthermore, DDP-resistant cells exhibited an increased capacity for DNA repair via the NHEJ pathway, with DNA-PKcs serving as a critical regulatory factor. Our data identified NHEJ activation as the primary driver of DDP resistance, with HR pathway inhibition showing no additive effect when combined with DNA-PKcs blockade.

Interestingly, while transcriptional upregulation of KU70 was observed, its protein level remained largely stable in resistant cells. This discrepancy may highlight a post-transcriptional regulatory layer, where the stability and function of KU70 are governed by its obligate heterodimerization with KU80 [35]. The pronounced overexpression of KU80 protein likely serves as the limiting factor that dictates the assembly of the functional KU complex, thereby positioning KU80 upregulation, rather than KU70, as the pivotal event driving NHEJ hyperactivation and consequent chemoresistance.

This study employed PDO models, which can preserve the heterogeneity of primary tumors and their microenvironmental characteristics and drug response profiles with high fidelity [36, 37]. This approach more realistically simulates drug resistance phenotypes in clinical practice. The use of PDO models effectively addresses the limitations of traditional cell lines regarding tumor heterogeneity and clinical relevance [38, 39], offering a more relevant experimental platform for elucidating the mechanisms underlying DDP resistance. Our results corroborated earlier findings indicating the essential function of DNA-PKcs in DNA repair and chemoresistance [39, 40]. However, unlike previous studies that focused on the canonical IKK/IκB pathway, we demonstrated that DNA-PKcs regulates NF-κB activation through p65 phosphorylation and acetylation, providing a novel mechanism for NF-κB signaling in CSCs. Through in vitro kinase assays, we confirmed that DNA-PKcs directly phosphorylates p65 at Ser536. Co-IP experiments also revealed that p300-mediated acetylation of p65 at Lys310 strictly relies on this phosphorylation event. This mechanistic cascade bypasses the canonical IKK/IκB pathway, offering a new therapeutic target for disrupting NF-κB-mediated stemness. Recent studies have underscored the role of DNA-PKcs in enhancing chemoresistance in several types of cancer, but its association with NF-κB activation and CSC formation in NSCLC received less attention [40–43]. Our study filled this gap by demonstrating that DNA-PKcs-mediated NF-κB activation is a key driver of DDP resistance in NSCLC. Bioinformatics analysis indicated that genes, such as WNT10A and WNT10B, are direct transcriptional targets of p65; however, their binding sites and functional relevance still need to be experimentally verified. In the future, the roles of these target genes in NF-κB-mediated stemness regulation can be clarified through ChIP-seq or gene editing techniques (e.g., CRISPR interference).

Our study revealed a novel mechanism of NF-κB activation, indicating that DNA-PKcs regulates p65 phosphorylation and acetylation, bypassing the canonical IKK/IκB pathway. This finding supports the notion that the function of nuclear p65 is regulated by these modifications [27]. DNA-PKcs serves as a marker for NHEJ and is a key signaling molecule that modulates post-translational modification of p65, thereby enhancing NF-κB activity. Acetylation of p65 at lysine 310 necessitates phosphorylation at serine 536, associated with RSK1 activation instead of IKK [28, 44, 45]. In A549/DR cells, p65 phosphorylation at S536 was associated with RSK1 overactivity, which was suppressed by DNA-PKcs knockdown in vitro and in vivo.

Cytokines in the tumor microenvironment can trigger NF-κB signaling by activating IKK [46, 47]. However, NF-κB activation generally lasts for a short while due to several negative feedback mechanisms [48]. Low IKK activity in A549 cells can be attributed to the fact that NF-κB signaling is sustained via p65 phosphorylation and acetylation, driven by DNA-PKcs activation in a positive feedback loop. Moreover, NU7441, a DNA-PKcs inhibitor, exhibited enhanced antitumor efficacy both in vivo and in vitro.

Our study offers new insights into the role of DNA-PKcs in DDP resistance and CSC formation. These findings align with precision oncology approaches, where computational methods like multi-view clustering [49] and bio-inspired optimization [50, 51] help decode complex biological systems (e.g., drug-resistant networks). Similar to IoT-fog-cloud workflows balancing efficiency and energy [52], these approaches can guide the design of targeted DNA-PKcs regimens by optimizing therapeutic efficacy and lowering toxicity. However, it is important to acknowledge several limitations. First, our findings were primarily based on in vitro experiments and animal models; therefore, further validation based on clinical data is needed. Secondly, our models did not fully replicate the involvement of tumor microenvironment, which plays a crucial role in chemoresistance. Future studies should explore the interplay between DNA-PKcs, NF-κB signaling, and the tumor microenvironment in DDP resistance. Additionally, the long-term effects of DNA-PKcs inhibition on normal tissue repair and immune response warrant further studies.

Beyond these scientific limitations, the clinical translation of our findings faces practical hurdles. First, the clinical sample size in this study was relatively limited due to the strict availability of patient tissues. Future validation with larger, independent patient cohorts will be essential to corroborate our findings and strengthen their clinical relevance. Secondly, the DNA-PKcs inhibitor used herein, NU7441, is a well-established research-grade compound but has not yet been approved for clinical use. Its translatability to human therapy therefore requires further investigation. Promisingly, several DNA-PK inhibitors (e.g., Nedisertib) are currently under evaluation in clinical trials (https://clinicaltrials.gov/). Future studies employing such clinically relevant inhibitors would help bridge the gap between preclinical findings and therapeutic applications.

## Conclusion

Our study revealed that DNA-PKcs induced NF-κB activation via p65 phosphorylation and acetylation, circumventing the traditional IKK/IκB pathway. NF-kB activation not only promoted DNA repair but also enhanced CSC traits, contributing to DDP resistance in NSCLC. Targeting DNA-PKcs with inhibitors, such as NU7441, not only enhanced DDP-induced DNA damage but also decreased CSC enrichment, offering a promising strategy to overcome chemoresistance.

## Supplementary Information

Below is the link to the electronic supplementary material.

Supplementary Material 1

## Abbreviations

DDP: Cisplatin
NSCLC: Non-small cell lung cancer
CSCs: Cancer stem cells
NHEJ: Non-homologous end joining
HR: Homologous recombination
DNA-PKcs: DNA-dependent protein kinase catalytic subunit
PDOs: Patient-derived organoids
PDOX: Patient-derived organoid-based xenografts
LCO: Lung cancer organoid
DSBs: DNA double-strand breaks
A549/DR: DDP-resistant derivatives
STR: Short tandem repeat
IHC: Immunohistochemical
