# Supplementary material for: Cisplatin-mediated activation of NF-κB promotes lung cancer stem cell formation via DNA repair pathways
Source: J Transl Med. 2025 Nov 21;23:1336. doi: 10.1186/s12967-025-07282-9 (PMC12639660; doi:10.1186/s12967-025-07282-9)
Supplement: Supplementary file 1 — Supplementary Material 1 [file 12967_2025_7282_MOESM1_ESM.docx]

**Supplementary Files**


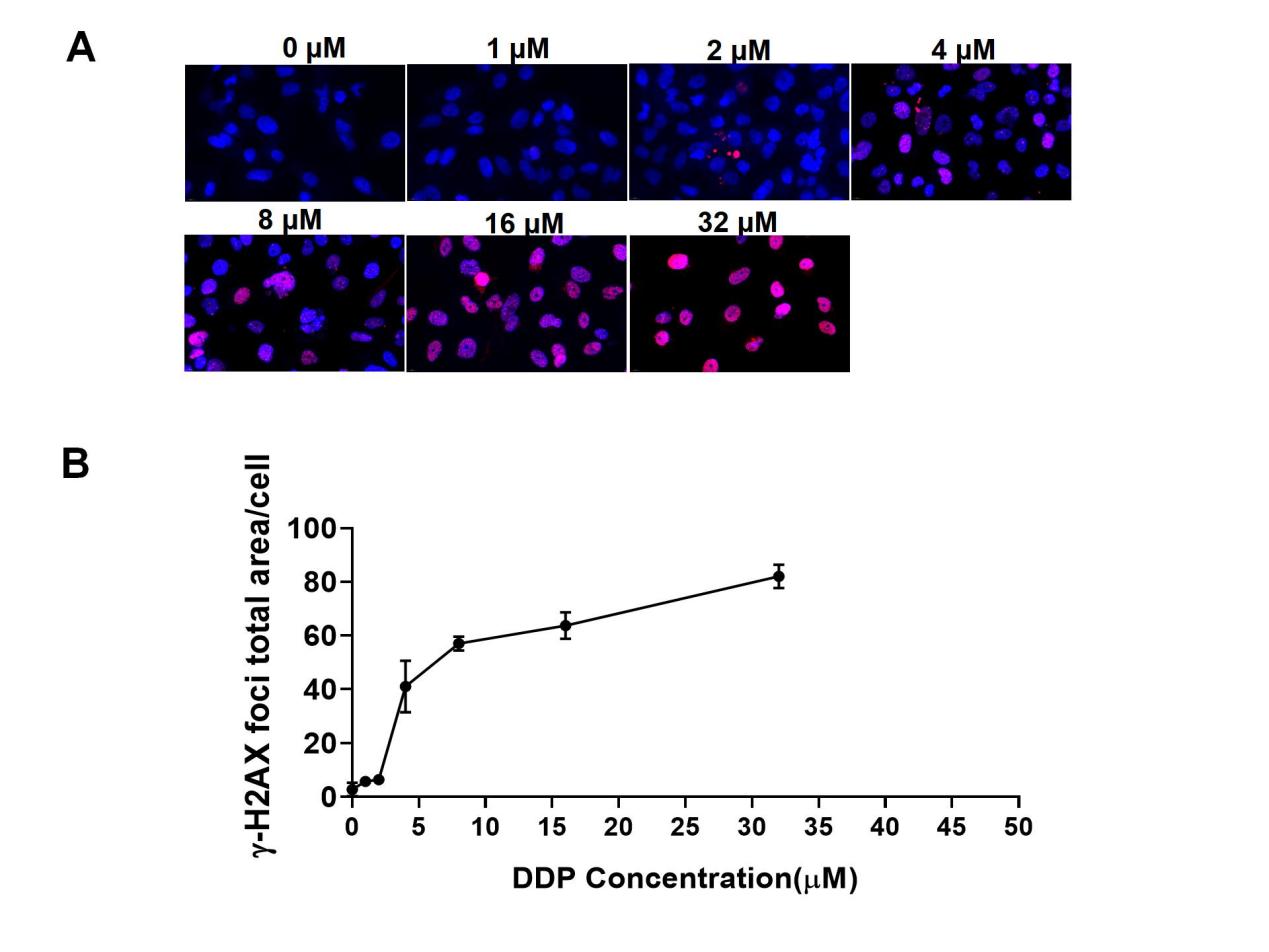


**Figure S1. The effect of DDP on DNA damage and repair in A549 Cells. A.** Fluorescence microscopy images depictingγ-H2AX foci (red) and cell nuclei (blue) were obtained after treatment with different concentrations of DDP(0, 1, 2, 4, 8, 16 and 32 μM) for 2 hours, with a magnification of 200x. **B** The graph illustrates the ratio of the total area of γ-H2AX foci to the cell area as a function of DDP concentration.


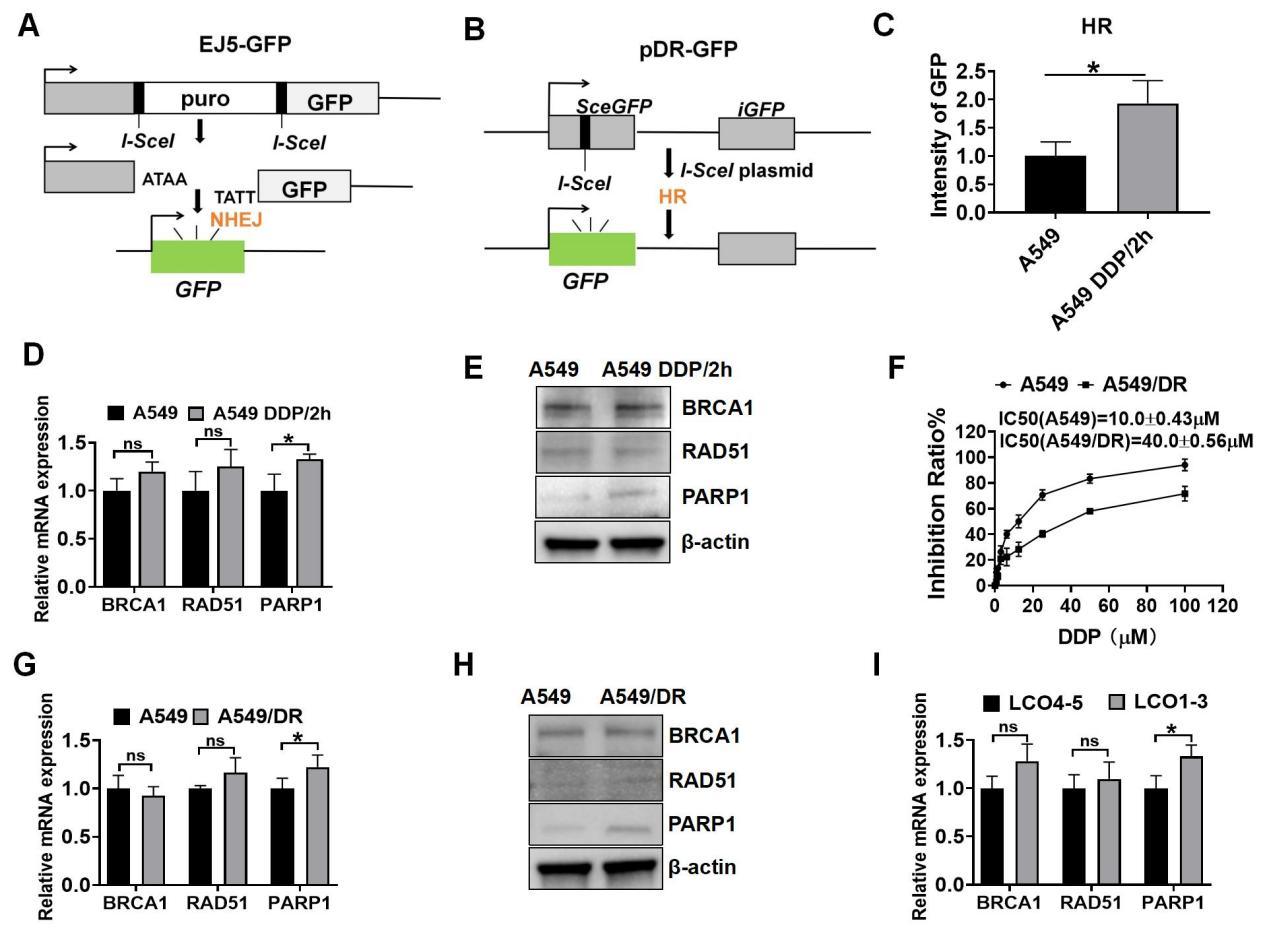


**Figure S2.**  **Effects of DDP on HR repair and NHEJ DNA repair gene expression in A549 cells.** **A-B** Schematic Representation of DNA Repair Mechanisms. **A** The diagram depicts HR repair using DR-GFP transgenes. **B** The diagram illustrates NHEJ repair employing EJ5-GFP transgenes obtained from Wu, L. (2013). Radiat Res, 179(2), 160−170. <https://doi.org/10.1667/RR3034.1.> **C** GFP intensity of A549 cells after the repair of I-SceI-induced DSBs via HR measured using DR-GFP assays. The cells were exposed to either a vehicle or 4 µM DDP. DDP/2h refers to A549 cells being stimulated with DDP for 2 hours, followed by drug removal. **D** Analysis of the expression of the HR gene in A549 cells using qRT-PCR. **E** Western blotting of BRCA1, RAD51, and PARP1 in A549 cells. **F** CCK8 assay for measuring cell proliferation in A549 and A549/DR cells 72 hours after exposure to DDP. **G** qRT-PCR was employed to measure the expression of HR genes in A549 and A549/DDP cells. **H** Western blotting was conducted to measure the expression of the related proteins in A549 and A549/DR cells. **I** qRT-PCR was conducted to measure the mRNA expression levels of BRCA1, RAD51, and PARP1 in LCOs. *P<0.05; ns, not significant.


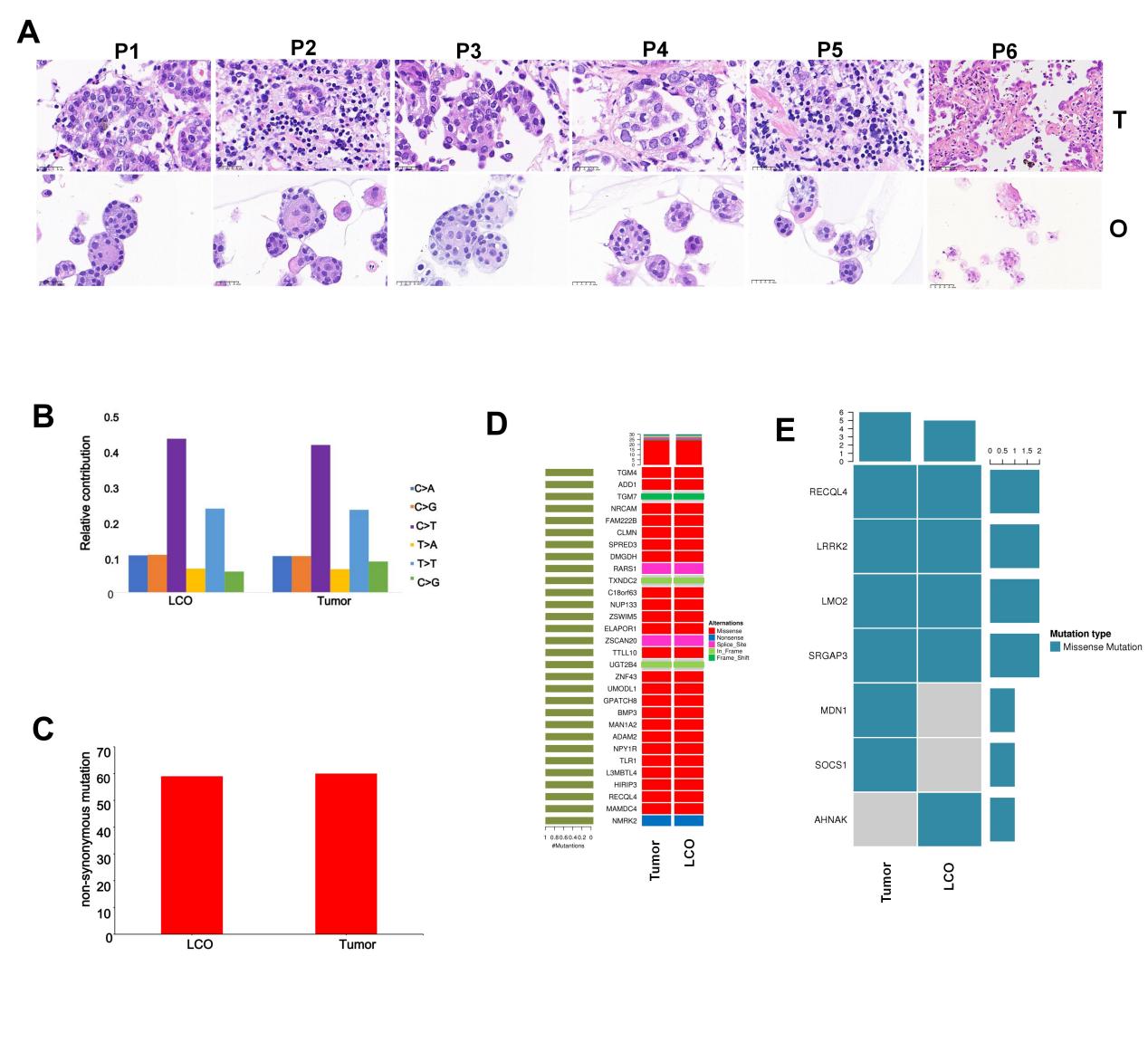


**Figure S3. Consistency verification of LCOs. A** HE staining for the morphological consistency of LCOs and paired tumor. O, LCOs; T, patients’ primary specimens, Scale bar = 25 μm. P1 to P6 represent NSCLC patients 1 to 6. **B** The incidence rates of various point mutations in the organoid cultures (LCOs) and their corresponding tumor samples**. C** The tumor mutational burden (TMB) was measured for the organoids and matched tumor tissues. **D** The high-frequency mutations in organoid lines (O) and the corresponding tissues (T). **E** Heatmap of CNVs in oncogenes. Gene copy numbers are transformed as log2 ratios per gene.


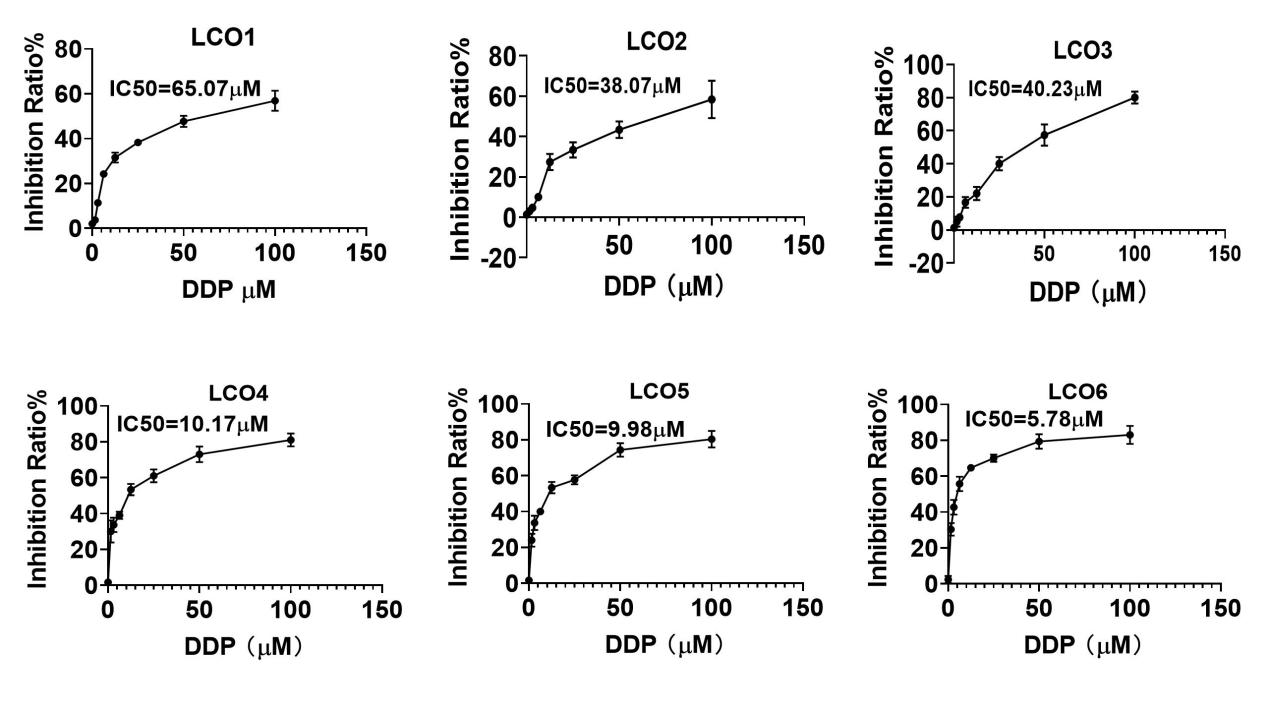


**Figure S4.** ATP assay analysis of the effect of 72-hour treatment with DDP on the viability of LCO1-6, and IC50 values for DDP in LCO1-6.


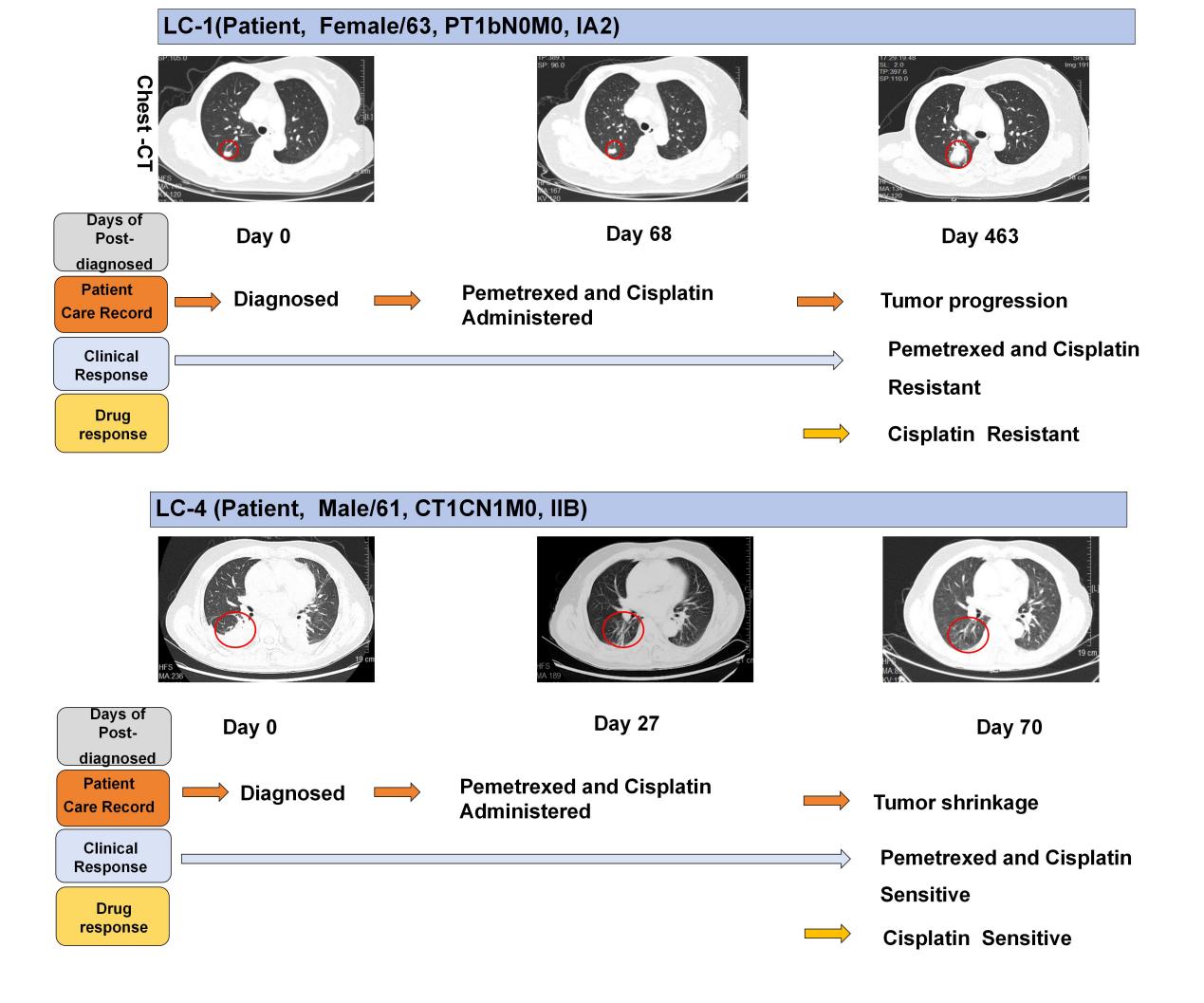


**Figure S5. Prediction of the clinical response to DDP using LCO models.** Patient #1 was diagnosed with stage IA2 lung cancer and experienced tumor progression after treatment with DDP. Thereafter, drug sensitivity test with LCO1 showed resistance to DDP. Patient #4 was diagnosed with stage IIB lung cancer and achieved significant lesion size reduction after treatment with DDP. Drug sensitivity tests revealed a strong positive response to DDP. The red circles in the images highlight the tumor areas before and after chemotherapy, as demonstrated by enhanced CT scans.


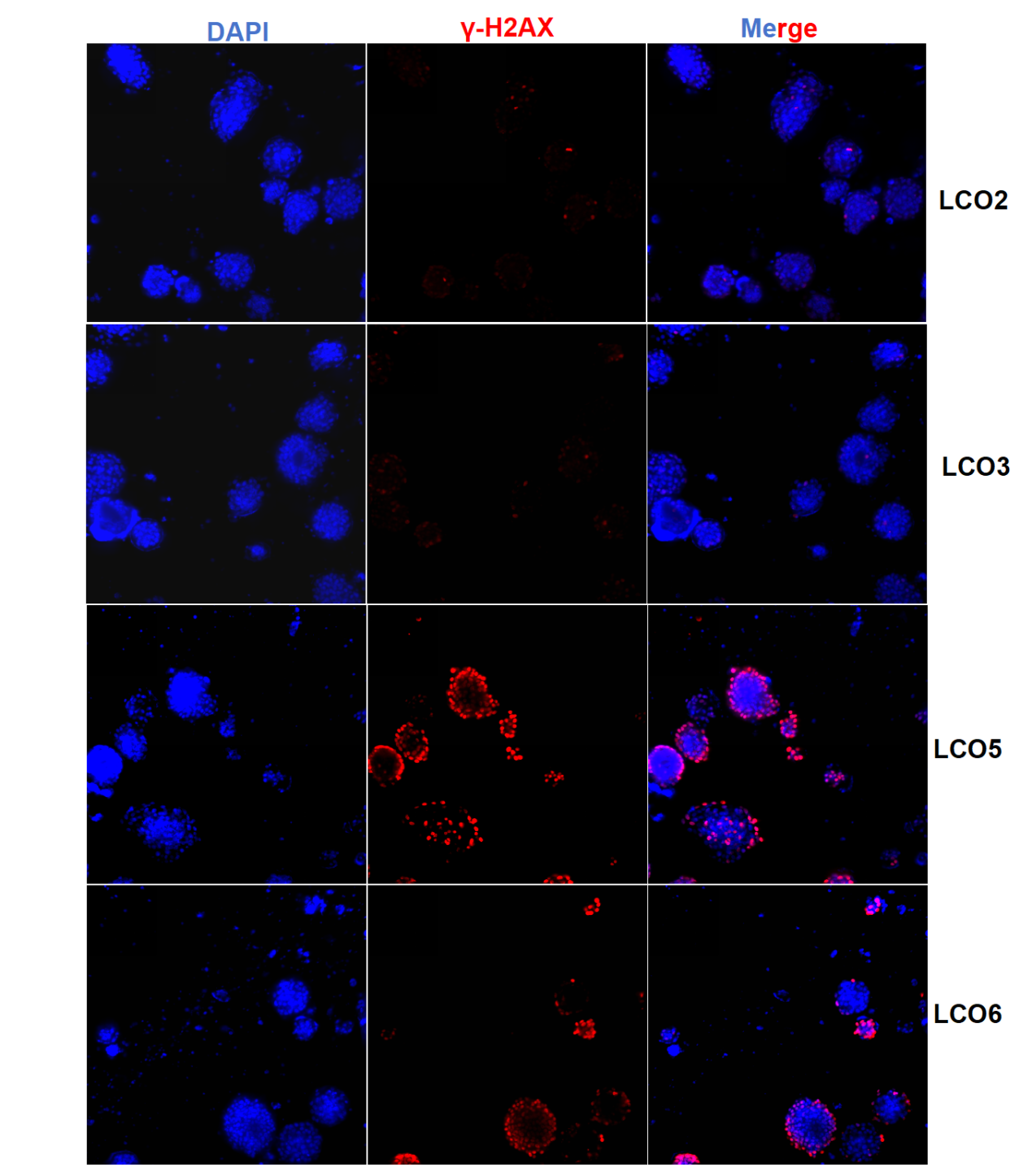


**Figure S6.** Immunohistochemical analysis of γ-H2AX expression in LCOs at a magnification of 200X. LCO2-3 were derived from DDP-resistant patients, while LCO5-6 from DDP-sensitive patients.


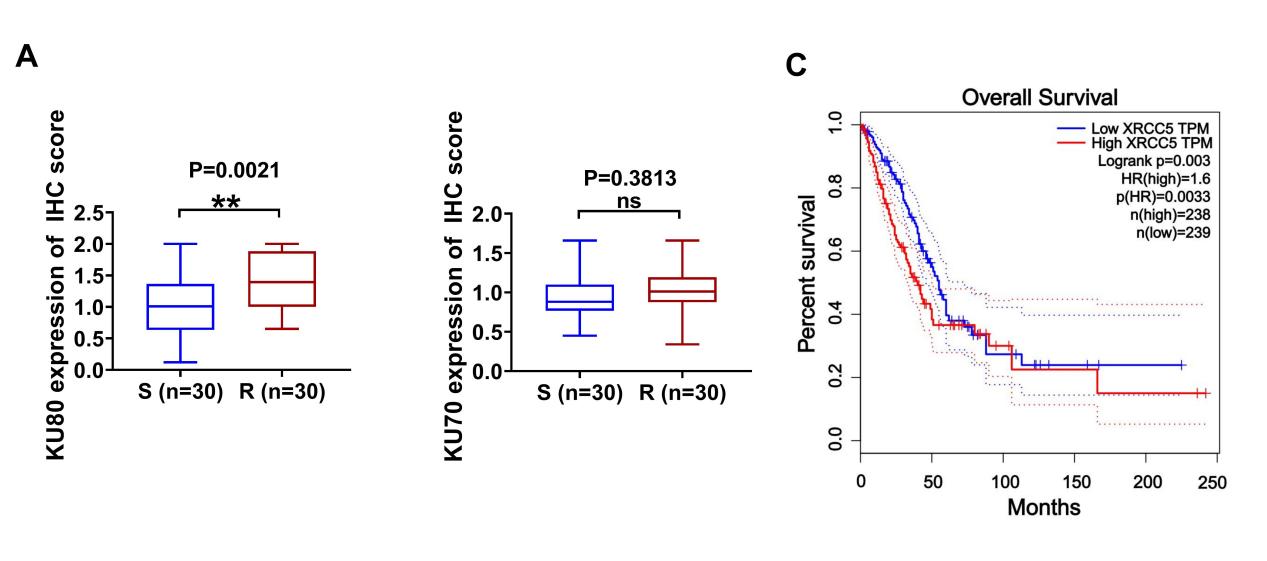


**Figure S7.** Association of KU80/kU70 expression with DDP resistance and clinical outcomes in lung cancer. **A** IHC intensity of KU70 and KU80 in DDP-sensitive tumors and DDP-resistant tumors (n=30). **B** In the Kaplan-Meier curve, the dotted line indicates the 95% CI. HR, hazard ratio. KU80, the protein product of the XRCC5 gene, which may not be directly searchable in some databases.


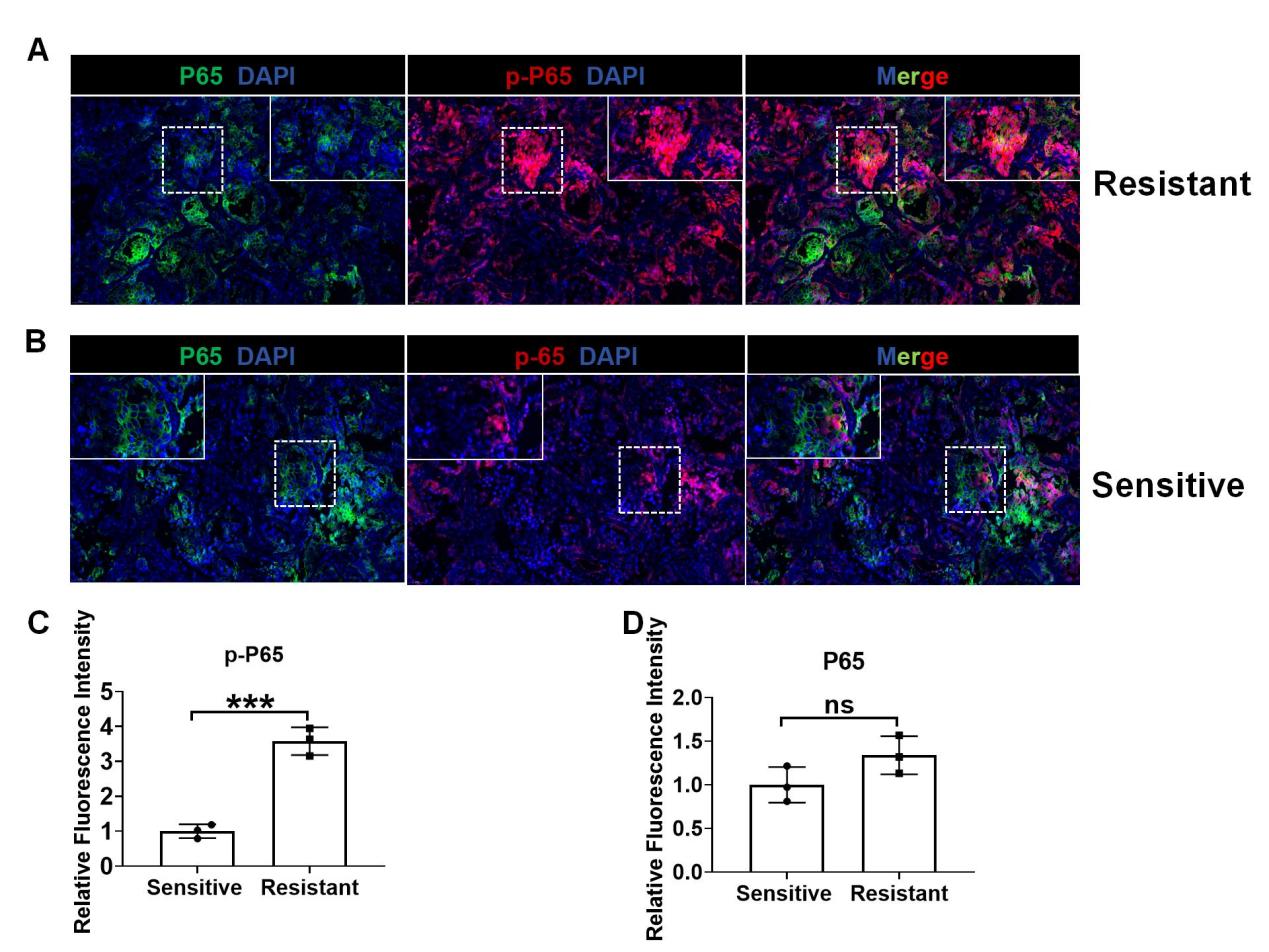


**Figure S8. Immunohistochemical Analysis of p-P65/P65 Expression in Treatment-Resistant and Treatment-Sensitive Lung Cancer. A-B** Representative immunohistochemical staining of p-P65 in lung cancer tissues, comparing treatment-resistant and treatment-sensitive cases (Scale bars: 200X). **C-D** Quantitative assessment of the expression levels of p-P65 (C) and total P65 (D), measured by relative fluorescence intensity. ***P<0.001; ns, not significant.


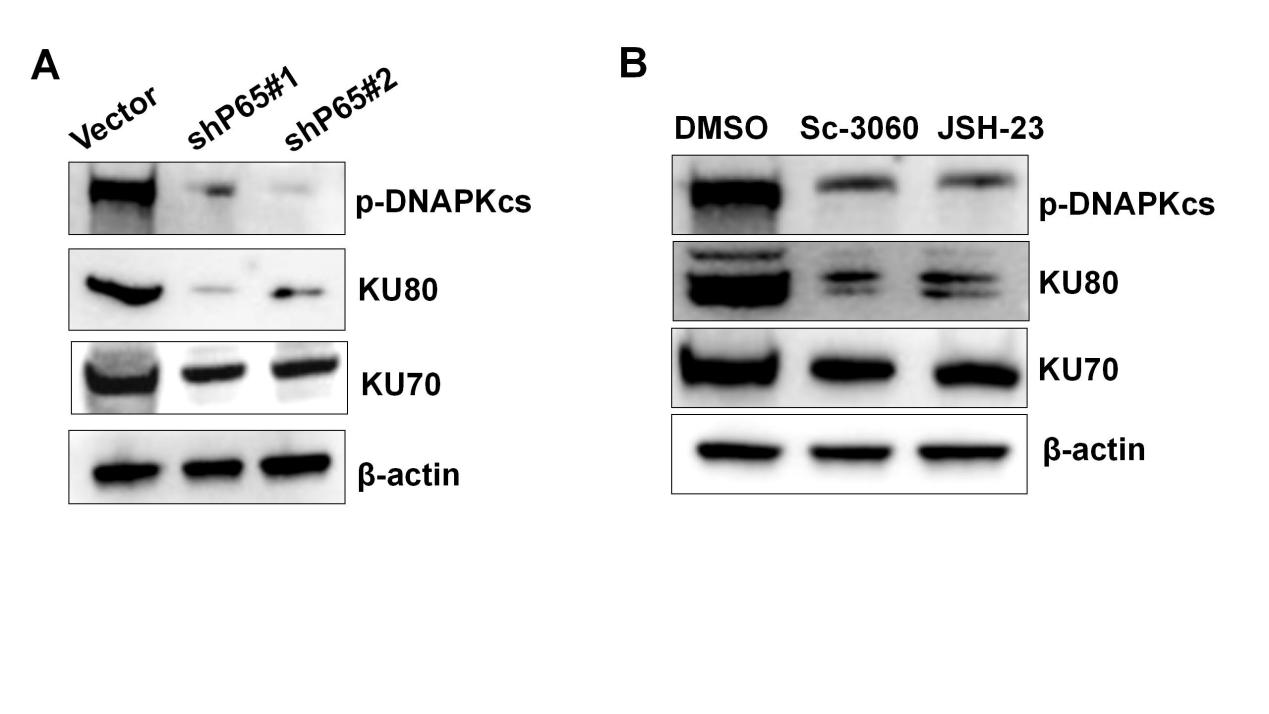
**Figure S9. Protein expression analysis by western blotting. A** Western blotting for KU70, KU80, and phosphorylated DNA-PKcs in A549/DR cells transduced with or without p65 shRNA. **B** Western blotting was conducted for KU70, KU80, and phosphorylated DNA-PKcs in samples pretreated with inhibitors of NF-kB nuclear translocation.


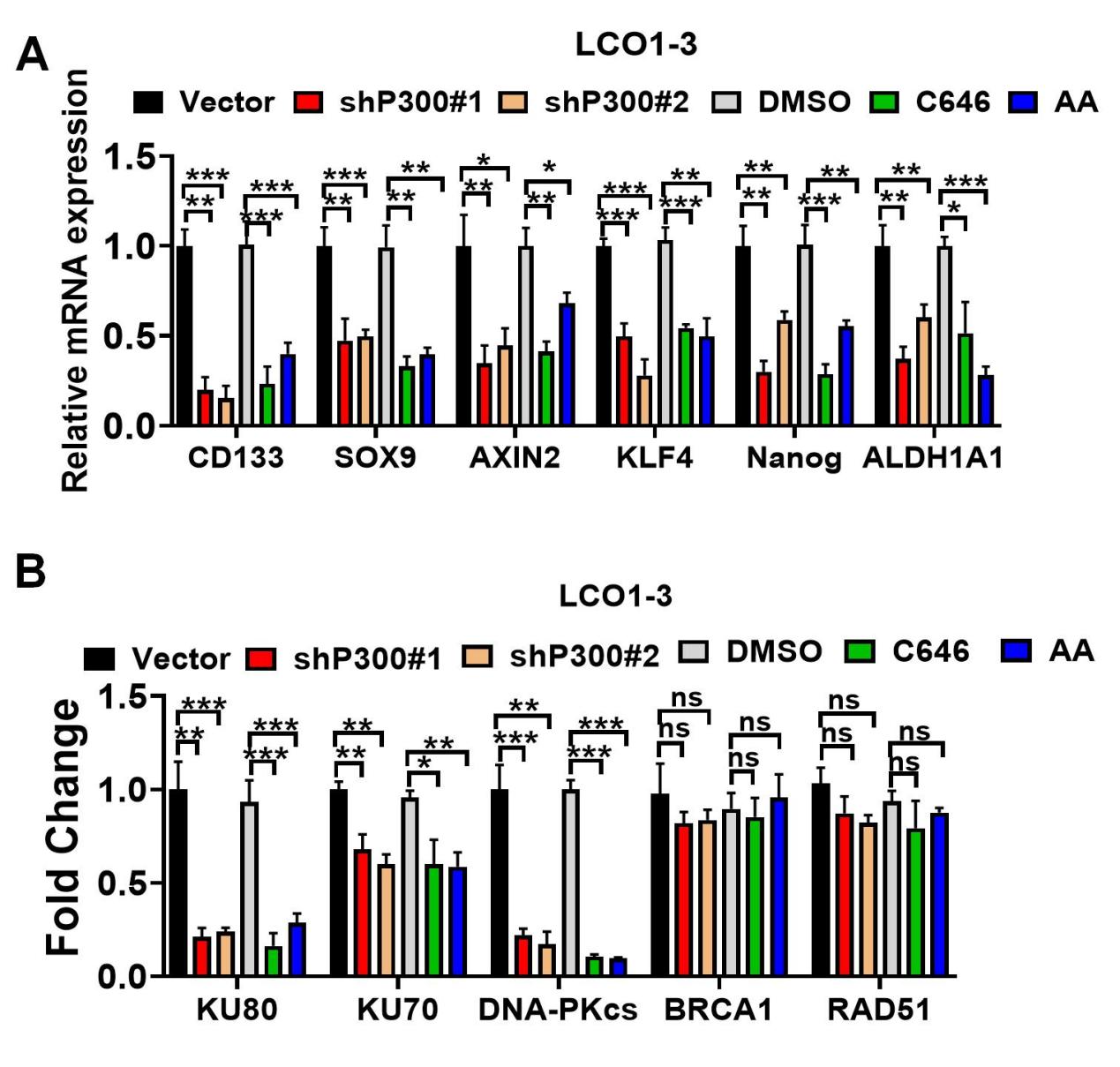


**Figure S10. RT-PCR was employed to measure the mRNA expression levels of stemness-related genes in LCO1-3(A) and DNA repair-related genes in LCO1-3(B).** LCOs cells were transduced either without (Vector) or with p300 shRNA, or pretreated with p300 inhibitors (C646 or anacardic acid [AA]), considering DMSO as the negative control. *P<0.05; **P<0.01; ***P<0.001; ns, not significant.


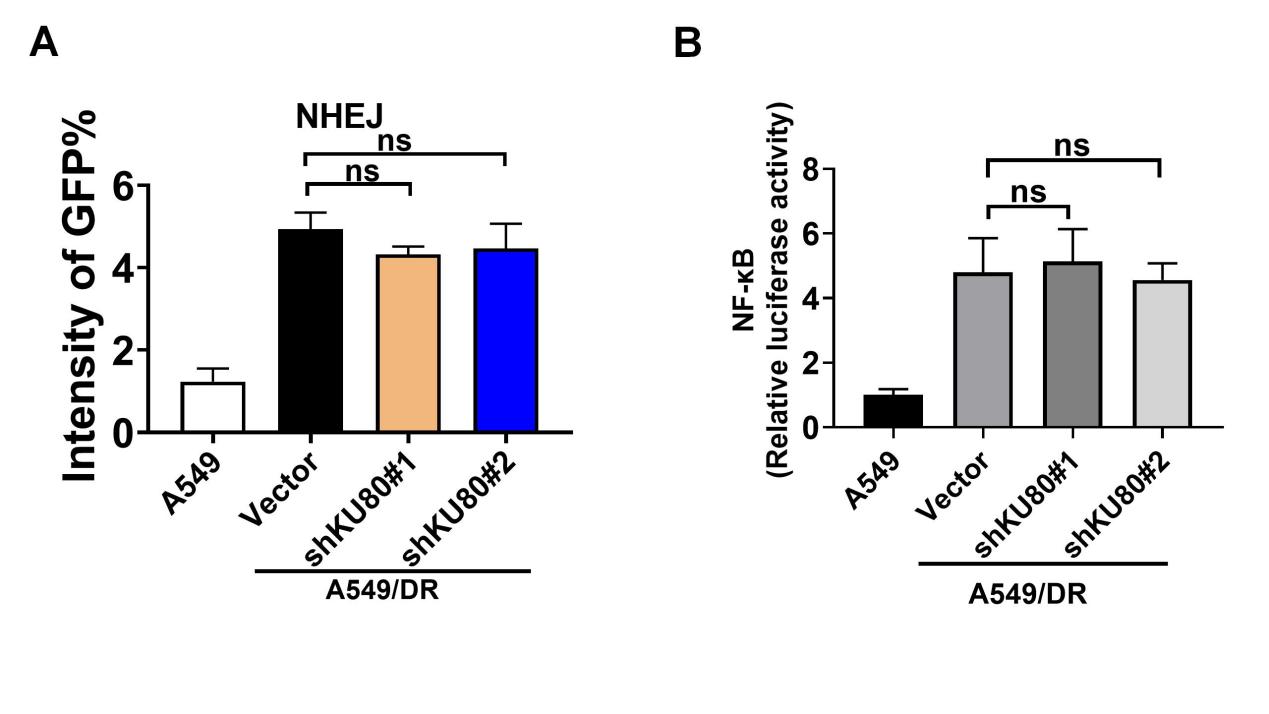


**Figure S11. The effect of KU80 on NHEJ and NF-κB activity.** A-B A549/DR cells were transduced with or without (vector) shRNA for KU80. **A** The intensity of GFP in OR cells repaired by NHEJ, measured using EJ5-GFP reporter assays. **B** Luciferase reporter assays showing NF-kB activity. ns, not significant.


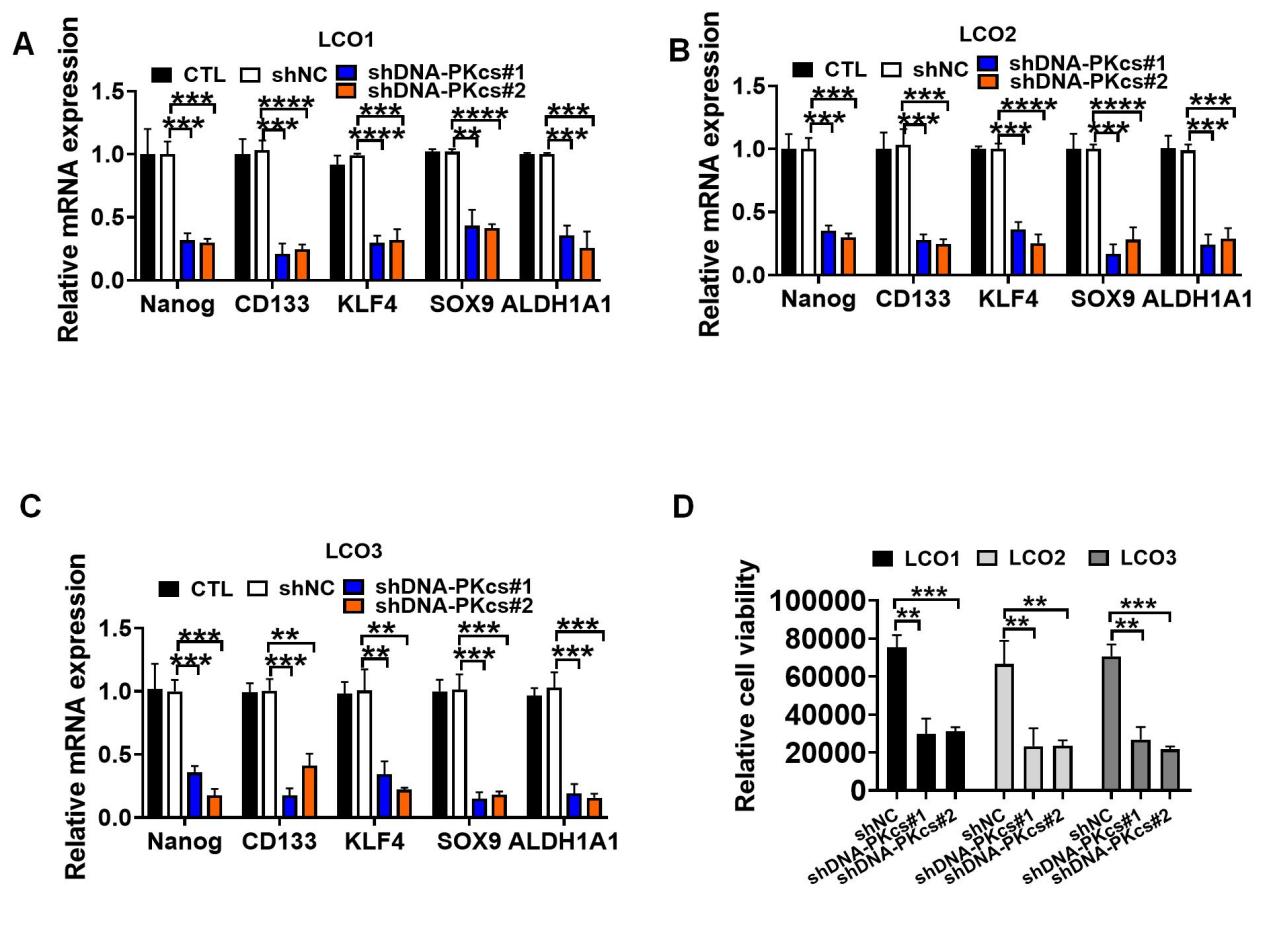


**Figure S12. DNA-PKcs downregulation reduces the mRNA expression levels of stemness genes and cell viability in LCO1-3 Cells.** **A-C** RT-PCR analysis of mRNA levels in LCO1 (A), LCO2 (B), and LCO3 (C) cells following DNA-PKcs downregulation. **D** Effect of DNA-PKcs downregulation on relative cell viability in LCO1-3 cells. **P<0.01; ***P<0.001.


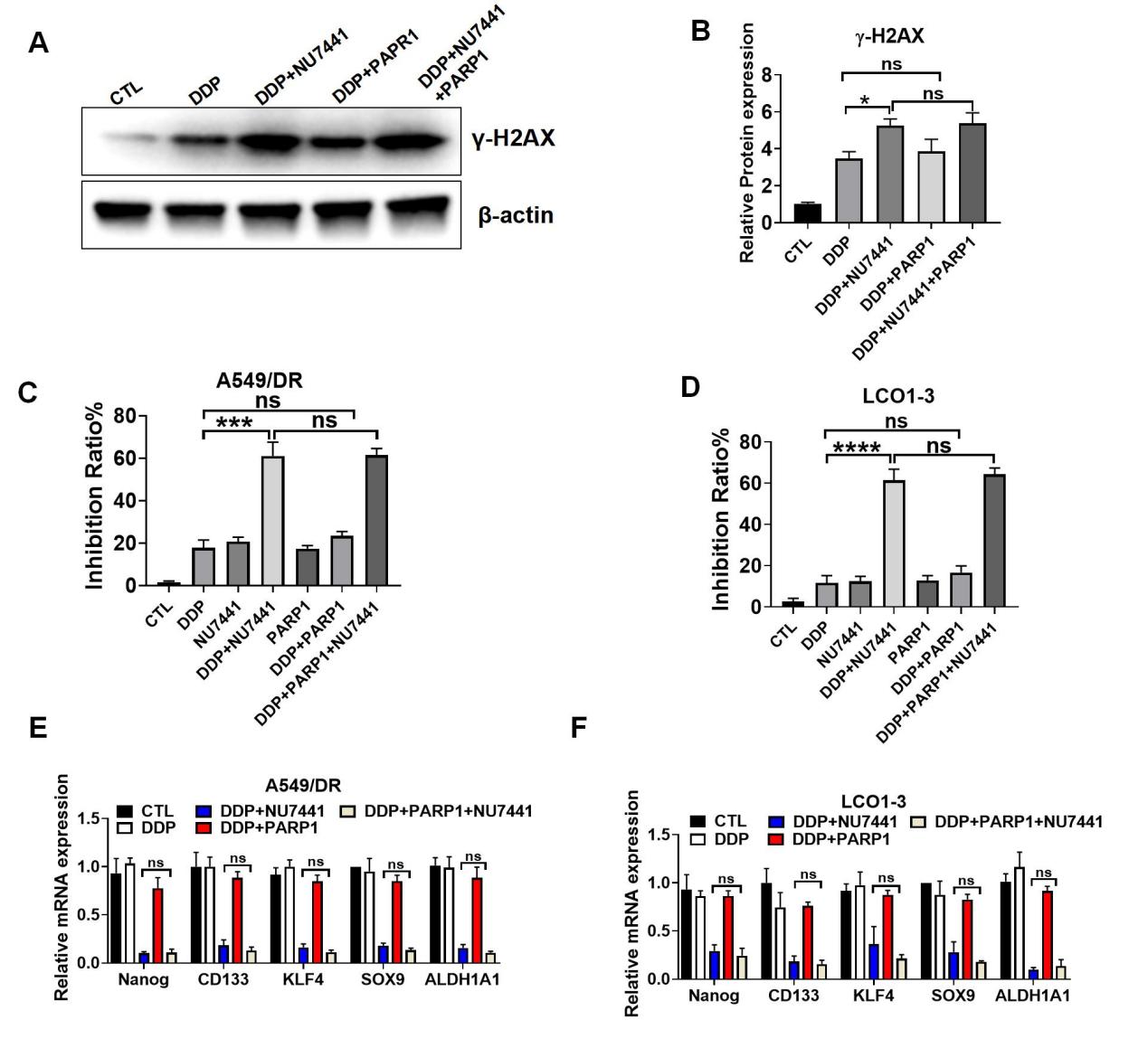


**Figure S13. HR pathway inhibition fails to enhance chemosensitivity or stemness suppression**. **A** γ-H2AX levels in A549/DR cells after treatment with/without PARP1 inhibition, measured by Western blotting. **B** The protein levels of γ-H2AX normalized to β-actin (n=3). **C-F** A549/DR cells were treated with vehicle control, 4 μM DDP, 4 μM DDP + 1 μM NU7441, 4 μM DDP + 10 μM PARP1 inhibitor, or the combination of all three agents. LCOs cells were treated with vehicle control, 10 μM DDP, 10 μM DDP + 2 μM NU7441, 10 μM DDP + 2 μM NU7441 +20 μM PARP1 inhibitor, or the combination of all three agents. **C-D** Growth inhibition rates of A549/DR (C）and LCO1 (D) cells**. E-F** Quantitative RT-PCR analysis of mRNA levels in A549/DR (E) and LCO1-3 (F) cells.


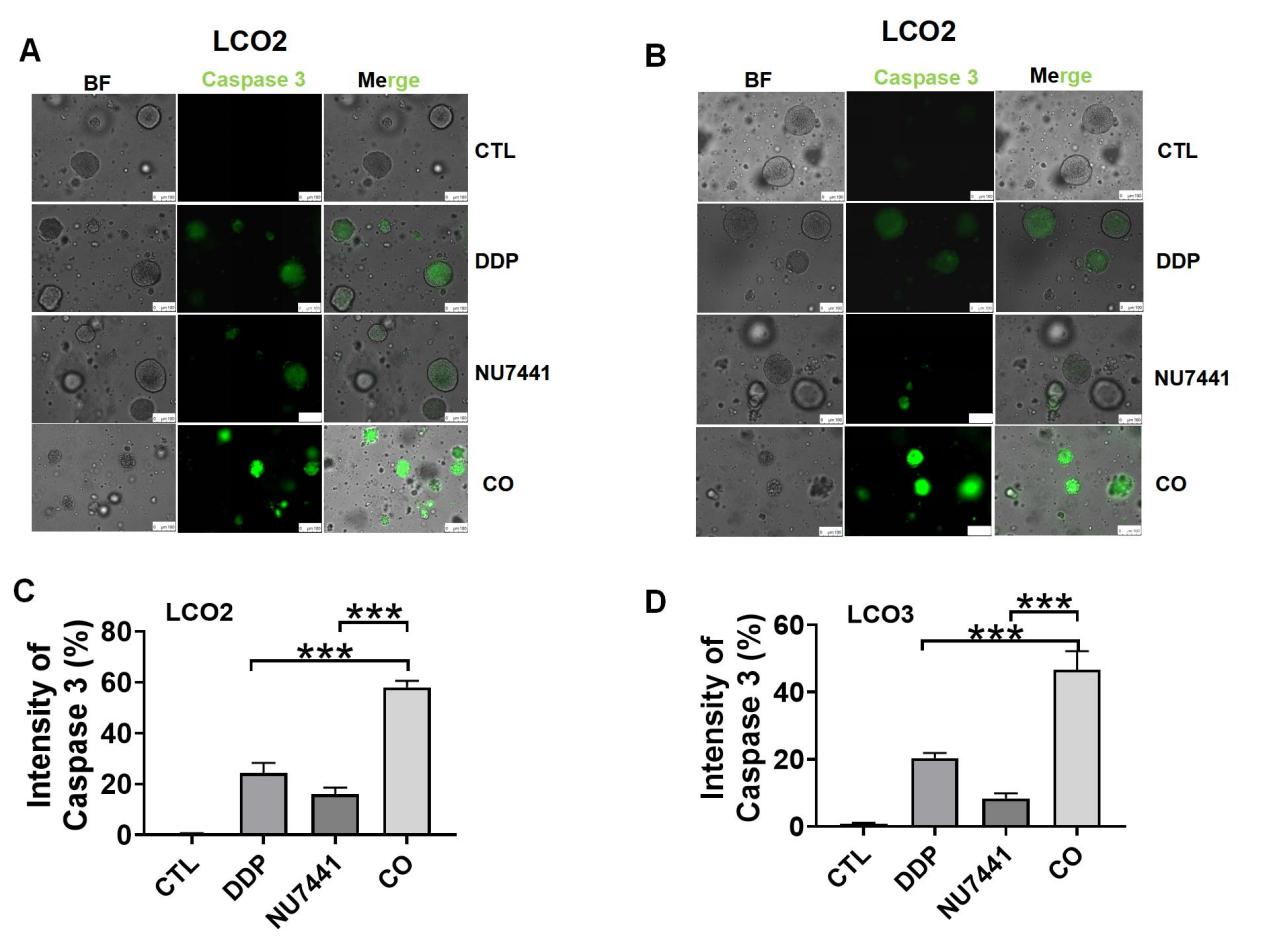


**Figure S14.** **Immunofluorescence assay indicated that treatment with NU7441 augmented caspase3 expression in LCO2-3 exposed to DDP.** **A-B** Representative immunofluorescence images of Caspase 3 cells in LCO2(A) and LCO3(B) (Scale bar = 100 μm); **C-D** Quantitative analysis of Caspase intensity in LCO2(C) and LCO3(B). CO, direct co-culture. ***P<0.001.

**Graphical Abstract**

**
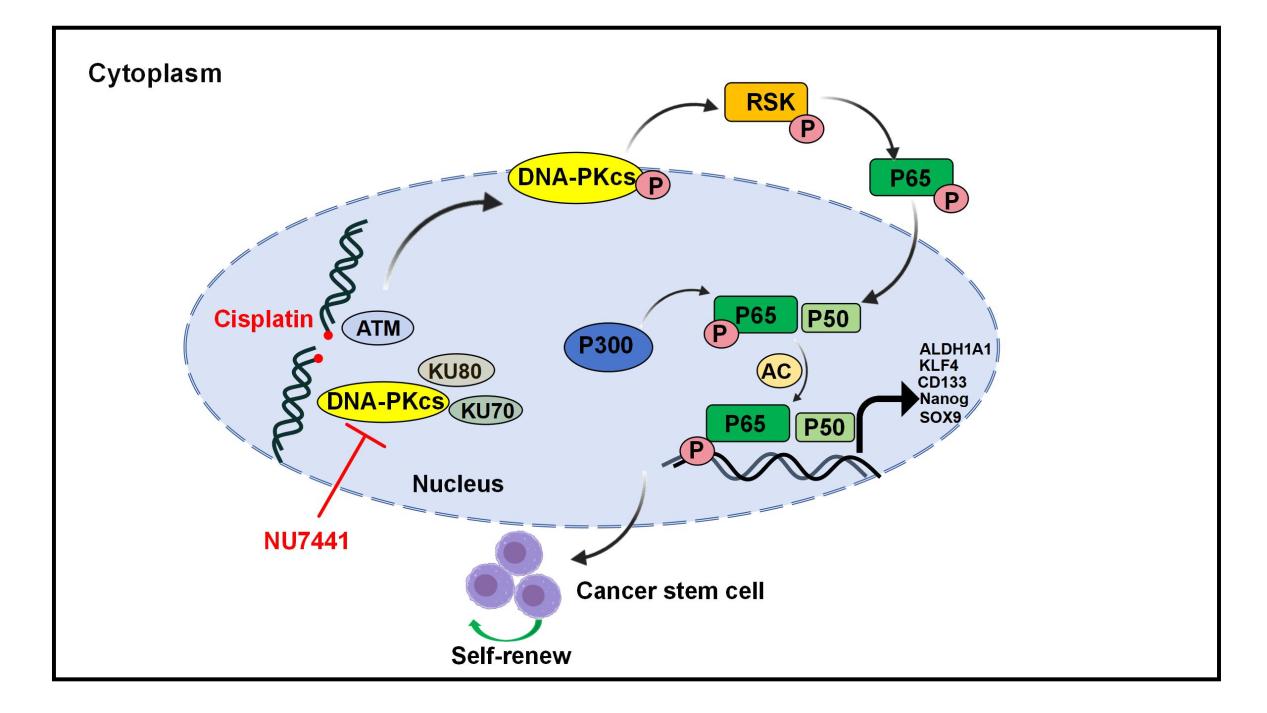
**

**Supplementary Table 1. The clinicopathological characteristics of the cases and LCOs subtype**

| Oranoid  line | SEX | Age | Tumor type | Tumor location | pT | pN | cM | stage | Response to DDP |
| --- | --- | --- | --- | --- | --- | --- | --- | --- | --- |
| LCO1 | Female | 63 | LC | Lung | pT1b | N0 | M0 | ⅠA2 | Resistance |
| LCO2 | Male | 76 | LC | Lung | pT2b | N2 | M0 | ⅢA | Resistance |
| LCO3 | Male | 59 | LC | Lung | cT4 | N0 | M0 | ⅢB | Resistance |
| LCO4 | Male | 61 | LC | Lung | T1 | N1 | M0 | ⅡB | Sensitive |
| LCO5 | Female | 67 | LC | Lung | pT1b | N0 | M0 | ⅠA2 | Sensitive |
| LCO6 | Male | 61 | LC | Lung | T4 | N0 | M1 | Ⅳ | Sensitive |
| 7 | Male | 58 | LC | Lung | cT4 | N0 | M0 | ⅢA | Resistance |
| 8 | Male | 78 | LC | Lung | cT1b | N2 | M1c | ⅣB | Sensitive |
| 9 | Male | 66 | LC | Lung | pT2b | N1 | M0 | ⅡB | Resistance |
| 10 | Male | 58 | LC | Lung | pT4a | N1 | M0 | ⅢA | Resistance |
| 11 | Female | 62 | LC | Lung | pT1 | N0 | M0 | ⅠA | Sensitive |
| 12 | Male | 70 | LC | Lung | pT3 | N0 | M0 | ⅡA | Sensitive |
| 13 | Male | 55 | LC | Lung | pT3 | N1b | M0 | ⅢB | Resistance |
| 14 | Male | 77 | LC | Lung | pT1b | N1 | M0 | ⅡB | Resistance |
| 15 | Male | 62 | LC | Lung | pT1a | N0 | M0 | ⅠA1 | Sensitive |
| 16 | Female | 71 | LC | Lung | cT3 | N2 | M0 | ⅡB | Sensitive |
| 17 | Male | 77 | LC | Lung | cT3 | N2b | M1b | ⅣA | Resistance |
| 18 | Female | 66 | LC | Lung | cT3 | N1 | M0 | ⅢA | Resistance |
| 19 | Male | 69 | LC | Lung | pT0 | N0 | M0 | ⅠA | Sensitive |
| 20 | Female | 73 | LC | Lung | cT1 | Nx | M1 | Ⅳ | Resistance |
| 21 | Male | 59 | LC | Lung | cT3 | N2 | M0 | ⅢB | Resistance |
| 22 | Male | 77 | LC | Lung | pT1 | CN1 | M0 | ⅡB | Sensitive |
| 23 | Male | 80 | LC | Lung | cT2a | N3 | M1 | ⅣA | Resistance |
| 24 | Female | 57 | LC | Lung | pT3 | N0 | M0 | ⅡB | Sensitive |
| 25 | Female | 63 | LC | Lung | pT3 | N1a | M0 | ⅢB | Resistance |
| 26 | Male | 59 | LC | Lung | pT2 | N2 | M0 | ⅢA | Resistance |
| 27 | Female | 69 | LC | Lung | cT2a | N2 | M0 | ⅢA | Resistance |
| 28 | Male | 72 | LC | Lung | T3 | N2 | M1 | Ⅳ | Resistance |
| 29 | Male | 71 | LC | Lung | pT2a | N2 | M0 | ⅢA | Sensitive |
| 30 | Female | 65 | LC | Lung | pT1b | N0 | M0 | ⅠA2 | Sensitive |
| 31 | Female | 66 | LC | Lung | cT4 | N2 | M1a | ⅣA | Resistance |
| 32 | Female | 68 | LC | Lung | cT2b | N1 | M0 | ⅡB | Sensitive |
| 33 | Female | 73 | LC | Lung | pT1 | N0 | M0 | ⅠA | Sensitive |
| 34 | Female | 57 | LC | Lung | cT2a | N1 | M0 | ⅡB | Sensitive |
| 35 | Male | 62 | LC | Lung | cT1a | N2 | M0 | ⅡA | Sensitive |
| 36 | Male | 67 | LC | Lung | cT2a | N1 | M0 | ⅡB | Resistance |
| 37 | Female | 59 | LC | Lung | pT2b | N0 | M0 | ⅡA | Sensitive |
| 38 | Female | 60 | LC | Lung | pT2a | N2 | M0 | ⅢA | Resistance |
| 39 | Female | 64 | LC | Lung | pT2 | N1 | M0 | ⅢA | Resistance |
| 40 | Female | 69 | LC | Lung | cT2a | N3 | M1 | Ⅳb | Resistance |
| 41 | Male | 71 | LC | Lung | pT1c | N0 | M0 | ⅠA3 | Sensitive |
| 42 | Male | 75 | LC | Lung | cT4 | N3 | M1 | IVA | Resistance |
| 43 | Male | 77 | LC | Lung | cT2b | N2 | M0 | ⅡA | Resistance |
| 44 | Male | 70 | LC | Lung | cT1b | N2 | M0 | ⅢA | Sensitive |
| 45 | Female | 60 | LC | Lung | pT2 | N0 | M0 | ⅢA | Resistance |
| 46 | Female | 63 | LC | Lung | cT4 | N0 | M0 | ⅢA | Sensitive |
| 47 | Male | 61 | LC | Lung | cT3 | N1 | M1 | Ⅳ | Sensitive |
| 48 | Female | 69 | LC | Lung | cT3 | N2 | M1 | ⅣB | \| Resistance \| \| --- \| |
| 49 | Male | 72 | LC | Lung | cT4 | N2 | M0 | ⅢB | Sensitive |
| 50 | Female | 50 | LC | Lung | pT3 | N1 | M1 | Ⅳ | Sensitive |
| 51 | Male | 56 | LC | Lung | pT1c | N0 | M0 | ⅠA3 | Sensitive |
| 52 | Male | 64 | LC | Lung | cT3 | N2 | M1 | ⅣB | Resistance |
| 53 | Male | 76 | LC | Lung | cT1b | N3 | M0 | ⅢB | Sensitive |
| 54 | Male | 55 | LC | Lung | cT2a | N3 | M1 | ⅣA | Resistance |
| 55 | Male | 77 | LC | Lung | cT2a | N3 | M1 | ⅣA | Sensitive |
| 56 | Male | 60 | LC | Lung | cT3 | N1 | M0 | Ⅲ | Sensitive |
| 57 | Male | 48 | LC | Lung | cT3 | N2 | M1 | ⅣB | Resistance |
| 58 | Female | 50 | LC | Lung | pT1 | N0 | M0 | ⅠA3 | Sensitive |
| 59 | Male | 66 | LC | Lung | cT2 | N0 | M0 | ⅠB | Sensitive |
| 60 | Male | 68 | LC | Lung | cT3 | N1 | M1 | Ⅳ | Resistance |
|  |  |  |  |  |  |  |  |  |  |

**Supplementary Table 2. The detailed information on antibodies**

| **Antibodies** | **Source** | **Application** |
| --- | --- | --- |
| P65 | Cell Signaling Technology (Cat #8242) | 1:1000 for WB  1:100 for IP  1:400 for IF |
| KU70 | Abcam (Cat #Ab92450) | 1:1000 for WB  1:100 for IHC |
| KU80 | Abcam (Cat #Ab80592) | 1:1000 for WB  1:250 for IHC |
| DNA-PKcs | Abcam (Cat #Ab32566) | 1:1000 for WB |
| p-DNA-PKcs | Abcam (Cat #Ab124918) | 1:5000 for WB  1:100 for IHC |
| γ-H2AX | Abcam (Cat #Ab81299) | 1:2000 for WB  1:200 for IF |
| Nanog | ABclonal(Cat #A22625) | 1:5000 for WB |
| ALDH1A1 | ABclonal(Cat #A1802) | 1:500 for WB |
| CD133 | ABclonal(Cat #A0219) | 1:1000 for WB |
| KLF4 | ABclonal(Cat #A13673) | 1:1000 for WB |
| β-actin | Abcam (Cat #Ab8146) | 1:5000 for WB |
| SOX9 | ABclonal(Cat #A19710) | 1:1000 for WB |
| p-IKKβ | Cell Signaling Technology (Cat #2697) | 1:1000 for WB |
| t-IKKβ | Cell Signaling Technology (Cat #2684) | 1:1000 for WB |
| p-IKBα | ABclonal(Cat #AP0707) | 1:500 for WB |
| t-IKBa | ABclonal(Cat #A19714) | 1:1000 for IHC |
| Caspase 3 | Cell Signaling Technology (Cat #9662) | 1:1000 for WB |
| Cleaved Caspase-3 | Cell Signaling Technology (Cat #9664) | 1:1000 for WB |
| PARP | Cell Signaling Technology (Cat #9542) | 1:1000 for WB |
| Cleaved PARP | Cell Signaling Technology (Cat #5625) | 1:1000 for WB |
| PARP | Cell Signaling Technology (Cat #9532) | 1:1000 for WB |
| Ac-p65（acetyl K310) | Abcam (Cat #Ab19870) | 1:1000 for WB |
| Methyl-p65 | Cell Signaling Technology(Cat#13188) | 1:1000 for WB |
| p-p65(S276) | Cell Signaling Technology (Cat #3037) | 1:1000 for WB |
| p-p65(S536) | Cell Signaling Technology (Cat #3033) | 1:1000 for WB  1:1000 for IF |
| RSK1 | Cell Signaling Technology (Cat #8408) | 1:1000 for WB |
| p-RSK1(Ser380) | Cell Signaling Technology (Cat #8308) | 1:1000 for WB |
| p300 | Proteintech(Cat #20695-1-AP) | 1:1000 for WB |
| GCN5 | Abcam (Cat #Ab321885) | 1:1000 for WB |
| PCAF | Santa Cruz Biotechnology(Cat #sc-13124) | 1:1000 for WB |
| P300 | Proteintech(Cat #20695-1-AP) | 1:1000 for WB |
| PARP1 | Abcam(Cat #A32138) | 1:1000 for WB |

**Supplementary Table 3: Target Sequences of lentivirus**

| **Name** | **Target sequences** |
| --- | --- |
| Negative Control Scramble | TTCTCCGAACGTGTCACGT |
| shKU80#1 | AGAAGAGGCATATTGAAATAT |
| shKU80#2 | AGTCAGCTGGATATTATAATT |
| shDNA-PKcs#1 | CACCTTACTCTGTTGAAATTA |
| shDNA-PKcs#2 | CAGCCCTGGACCTTCTTATTA |

**Supplementary Table 4: The sequences of primers for RT-qPCR**

| Genes | Accession # | Sequences of Primers |
| --- | --- | --- |
| GAPDH | NM_002046.7 | Forward: 5’-AAGGTGAAGGTCGGAGTCAAC-3’  Reverse: 5’-GGGGTCATTGATGGCAACAATA-3 |
| KLF4 | [NM_001314052.2](https://www.ncbi.nlm.nih.gov/nuccore/NM_001314052.2) | Forward: 5’-TGACTGGGACGGCTGTGGATG-3’  Reverse: 5’-CTTCATGTGTAAGGCGAGGTGGTC-3’ |
| EPCAM | [NM_002354.3](https://www.ncbi.nlm.nih.gov/nuccore/NM_002354.3) | Forward: 5’-AAGGCCAAGCAGTGCAACGG-3’  Reverse: 5’-TCCAGTAGGTTCTCACTCGCTCAG-3’ |
| SOX9 | NM_000346.4 | Forward: 5’-CACACGCTGACCACGCTGAG-3’  Reverse: 5’-GCTGCTGCTGCTCGCTGTAG-3’ |
| CD133 | NM_001145847.2 | Forward: 5’-GTGGCGTGTGCGGCTATGAC-3’  Reverse:5’-CCAACTCCAACCATGAGGAAGACG-3’ |
| ALDH1A1 | NM_000689.5 | Forward:5’-ACGCCAGACTTACCTGTCCTACTC-3’  Reverse:5’-TCTTGCCACTCACTGAATCATGCC-3’ |
| NANOG | NM_001297698.2 | Forward:5’-AGATGCCTCACACGGAGACTG-3’  Reverse:5’-GGGTTGTTTGCCTTTGGGACTG-3’ |
| KU70 | NM_001288976.2 | Forward:5’-GTGCTCTGCCATCAAGTGTCTG  Reverse:GGTCACCAACTCTTCTTCCTGTG |
| KU80 | [NM_021141.4](https://www.ncbi.nlm.nih.gov/nuccore/NM_021141.4) | Reverse:GTGCTCTGCTCATCAAGTGTCTG  Forward:GGTCATCCAACTCTTCTTCCTGTG |
| DNA-PKcs | NM_001081640.2 | Reverse:AGTGAGCCAGCCTGCCTTG  Forward:CACCTTCTCTGAATCCTCTGAACTG |
| RAD51 | NM_001164269.2 | Reverse:TGGCAGTGGCTGAGAGGTATG  Forward: GGTCTGGTGGTCTGTGTTGAAC |
| ATM | NM_000051.4 | Forward:AGAGATTGTGGTGGAGTTATTGATGAC  Reverse:ATGAGGTGGATTAGGAGCAGGATC |

**Supplementary Table 5. Correlation between p-DNA-PKcs expression and clinicopathological characteristics in 60 patients with GC**

| Variables | Cases | p-DNA-PKcs expression | | P |
| --- | --- | --- | --- | --- |
|  |  | High  (≥median) | Low  (<median) |  |
| Number | 60 | 30 | 30 |  |
| Age |  |  |  | 0.8433 |
| <60 | 8 | 5 | 3 |  |
| ≥60 | 52 | 27 | 25 |  |
| Sex |  |  |  | 0.8566 |
| Female | 20 | 8 | 12 |  |
| Male | 40 | 22 | 18 |  |
| Lymph node metastasis |  |  |  | 0.0188 ***** |
| Yes | 31 | 21 | 10 |  |
| No | 29 | 9 | 29 |  |
| Distal metastasis |  |  |  | 0.0061 ****** |
| Yes | 18 | 10 | 8 |  |
| No | 42 | 18 | 24 |  |
| TNM stage |  |  |  | 0.0495 ***** |
| Ⅰ | 12 | 2 | 10 |  |
| Ⅱ | 13 | 3 | 10 |  |
| Ⅲ | 21 | 11 | 10 |  |
| Ⅳ | 14 | 10 | 4 |  |

*P < 0.05; **P < 0.01.

**Supplementary Table 6: JASPAR-based Prediction of Transcription Factor Binding Sites and Target Genes**

| TF  Gene  id | TF gene name | Target  gene  id | Target gene name | Pvalue | Start | Stop | Matched sequence |
| --- | --- | --- | --- | --- | --- | --- | --- |
| ENSG00000173039 | RELA | ENSG00000104332 | SFRP1 | 9.54E-7 | 373 | 382 | GGGAATTTCC |
| ENSG00000173039 | RELA | ENSG00000135925 | WNT10A | 9.54E-7 | 384 | 393 | GGGAATTTCC |
| ENSG00000173039 | RELA | ENSG00000122861 | PLAU | 9.54E-7 | 583 | 592 | GGGAATTTCC |
| ENSG00000173039 | RELA | ENSG00000100968 | NFATC4 | 2.86E-6 | 481 | 490 | GGGAGTTTCC |
| ENSG00000173039 | RELA | ENSG00000075651 | PLD1 | 6.68E-6 | 591 | 600 | GGGGGTTTCC |
| ENSG00000173039 | RELA | ENSG00000115266 | APC2 | 7.63E-6 | 457 | 466 | CGGGATTTCC |
| ENSG00000173039 | RELA | ENSG00000169884 | WNT10B | 9.57E-6 | 453 | 465 | TGGGGAAGCCCCA |
